# Factors during Production of Cereal-Derived Feed That Influence Mycotoxin Contents

**DOI:** 10.3390/toxins14050301

**Published:** 2022-04-25

**Authors:** Yvette Hoffmans, Sara Schaarschmidt, Carsten Fauhl-Hassek, H.J. van der Fels-Klerx

**Affiliations:** 1 Wageningen Food Safety Research, Akkermaalsbos 2, 6708 WB Wageningen, The Netherlands; yvette.hoffmans@wur.nl; 2 Department Safety in the Food Chain, German Federal Institute for Risk Assessment (BfR), Max-Dohrn-Str. 8-10, D-10589 Berlin, Germany; sara.schaarschmidt@bfr.bund.de (S.S.); carsten.fauhl-hassek@bfr.bund.de (C.F.-H.)

**Keywords:** processing, influential factors, aflatoxin, deoxynivalenol, fumonisin, HT-2, T-2, zearalenone, grains

## Abstract

Mycotoxins are naturally present in cereal-based feed materials; however, due to adverse effects on animal health, their presence in derived animal feed should be minimized. A systematic literature search was conducted to obtain an overview of all factors from harvest onwards influencing the presence and concentration of mycotoxins in cereal-based feeds. The feed production processes covered included the harvest time, post-harvest practices (drying, cleaning, storage), and processing (milling, mixing with mycotoxin binders, extrusion cooking, ensiling). Delayed harvest supports the production of multiple mycotoxins. The way feed materials are dried after harvest influences the concentration of mycotoxins therein. Applying fungicides on the feed materials after harvest as well as cleaning and sorting can lower the concentration of mycotoxins. During milling, mycotoxins might be redistributed in cereal feed materials and fractions thereof. It is important to know which parts of the cereals are used for feed production and whether or not mycotoxins predominantly accumulate in these fractions. For feed production, mostly the milling fractions with outer parts of cereals, such as bran and shorts, are used, in which mycotoxins concentrate during processing. Wet-milling of grains can lower the mycotoxin content in these parts of the grain. However, this is typically accompanied by translocation of mycotoxins to the liquid fractions, which might be added to by-products used as feed. Mycotoxin binders can be added during mixing of feed materials. Although binders do not remove mycotoxins from the feed, the mycotoxins become less bioavailable to the animal and, in the case of food-producing animals, to the consumer, lowering the adverse effects of mycotoxins. The effect of extruding cereal feed materials is dependent on several factors, but in principle, mycotoxin contents are decreased after extrusion cooking. The results on ensiling are not uniform; however, most of the data show that mycotoxin production is supported during ensiling when oxygen can enter this process. Overall, the results of the literature review suggest that factors preventing mycotoxin production have greater impact than factors lowering the mycotoxin contents already present in feed materials.

## 1. Introduction

Mycotoxins are secondary metabolites produced by fungi that can exert adverse effects on animals after exposure via feed consumption. A large study on both feed and feed materials from 100 countries all over the world showed that 88% of the samples contained at least one mycotoxin [1]. In the case of animals used for food production, (part of the) mycotoxins present in animal feed can be carried over along the feed–food chain to the consumers [2]. For example, aflatoxin B1 (AFB1) is carcinogenic for humans [3], and transfer of AFB1 into food products from animal origin is possible. Therefore, the presence of AFB1 in feed should be prevented. Due to adverse health effects in animals (shown in Table 1), the presence of mycotoxins in feed is undesired and should be as low as possible. The occurrence of adverse effects in animals is dose-dependent, which can differ per mycotoxin and per animal species and/or age class [4,5,6,7,8,9,10,11,12]. As an example, no effect was observed with regards to vulva appearance and uterus weight in piglets at a dose of 10.4 µg/kg body weight/day of zearalenone (ZEN), while a no observed adverse effect level (NOAEL) of 40 µg/kg body weight/day was established for ZEN in mature female pigs based on prolonged cycling [11]. Pigs are sensitive to the effect of deoxynivalenol (DON) on their reproduction [10], and feed producers try to produce complete feeds for pigs with the lowest possible DON concentration. Toxicity data, such as dose–response data, form the foundation for maximum levels set in legislative acts in order to protect animal and human health from the adverse effects of mycotoxins. The European Commission has established, with Commission Regulation (EC) No 1881/2006 [13], maximum levels in food products and their raw materials. Maximum levels for the mycotoxins AFB1 and ergot alkaloids in feed products and their commodities have been set via Directive 2002/32/EC [14], and guidance values for several mycotoxins in feed materials have been set in Commission Recommendation 2006/576/EC [15]. However, given the large effects of the environment (mainly local weather) on the presence of causative fungi and their production of mycotoxins in crops, it remains a challenge to limit the natural occurrence of mycotoxins, despite the efforts and the principle of as-low-as-reasonable-achievable taken by stakeholders.

The majority of mycotoxins are thermally stable contaminants, and heating of feed materials can hardly reduce their concentrations therein [4,5,6,7,8,9,10]. However, during the production of feed, processing leads to dilution of mycotoxin concentrations and/or redistribution of mycotoxins in the different fractions of the processed product. During feed production, processing can be used to lower mycotoxin concentrations in finished products for specific (sensitive) animal species. For this reason, it is important to be aware of the factors that influence the presence and levels of mycotoxins during feed production. In this way, an increase in mycotoxin concentrations during feed processing can be prevented, and mycotoxin concentrations in feed can be held as low as reasonably achievable.

The aim of this study was to investigate influential factors for mycotoxin presence during the production of cereal-based feeds. Based on a literature review, this paper presents an extensive overview of the feed production processes, from harvest until end-product and their effects on the contents of mycotoxins in the processed cereal products.

Major raw materials for complete and compound feed production in Europe include the cereals wheat, maize and barley, rye, and triticale [16]. Besides the raw materials, cereal-based derived products, such as silage, are often used as feed [17]. The processes of feed production, such as harvest, post-harvest practices (drying, cleaning, storage), and processing (milling, mixing with mycotoxin binders, extrusion cooking, ensiling) are presented in this paper. The mycotoxins included in this research are aflatoxins, DON, ergot alkaloids, fumonisins (FUM), ochratoxin A (OTA), T-2 toxin, HT-2 toxin, and ZEN. This selection of mycotoxins is based on the fact that there are legal instruments present within the European Union (EU) for these mycotoxins in feed. Albeit maximum limits in feed are only set for AF and rye ergot alkaloids, the EC has given recommendations for the maximum levels in feed for the other mycotoxins mentioned. An overview of the selected mycotoxins, their adverse effects on animals, the mycotoxin-producing fungi, and the EU legal instruments with the respected values are presented in Table 1.

**Table 1:** Mycotoxins, their fungal producers, the adverse effects to animal health, and legal limits or guidance values for their presence in cereals and derived animal feeds in the EU.

Mycotoxin(s)	Producers	Adverse Effects on Animals	EU Legal Instrument	Value (mg/kg ^1^)
Aflatoxin B1 (AFB1)	*Aspergillus* spp., mainly *Aspergillus flavus, Aspergillus parasiticus* [4]	Centrolobular necrosis, bile duct proliferation, kidney lesions [4]	Maximum limit	0.02 (feed materials, compound feed for the following animals except dairy animals and young animals: cattle, sheep, goats, pigs, poultry), 0.01 (complementary and complete feed other than the before and below mentioned), 0.005 (compound feed for dairy and young animals) [14]
Aflatoxin B2 (AFB2), Aflatoxin G1 (AFG1), Aflatoxin G2 (AFG2)	*Aspergillus* spp., mainly *Aspergillus flavus, Aspergillus parasiticus* [4]	Similar to AFB1 [4]	n.a.^2^	n.a.
Deoxynivalenol (DON)	*Fusarium* spp. [10]	Reduced body weight, reduced feed intake, feed conversion reduction, reproduction disorders, vomiting [10]	Guidance value	8 (feed materials of cereals and cereal products other than maize by-products), 12 (feed materials of maize by-products), 5 (compound feed other than the following), 0.9 (compound feed for pigs), 2 (compound feed for calves, lambs (young sheep), kids (young goat), and dogs) [15]
Ergot alkaloids	*Claviceps* spp. [9]	Reduced body weight, lameness, ill thrift, reduced feed intake, reduced heart weight, duodenum inflammations [9]	Maximum limit	1000 (feed materials and compound feed containing unground cereals) [14]
Fumonisin B1 (FB1), fumonisin B2 (FB2)	*Fusarium* spp. [7]	Lung weight increase, pulmonary oedema, hydrothorax, hepatic necrosis, cholestasis, encephalitis, hepatic nodular hyperplasia, alterations in several serum biochemical parameters [7]	Guidance value for FB1 + FB2	60 (feed materials of maize and maize products), 5 (compound feed for pigs, horses, rabbits, and pet animals), 10 (compound feed for fish), 20 (compound feed for poultry, calves, lambs, and kids), 50 (compound feed for adult ruminant and mink) [15]
Ochratoxin A (OTA)	*Aspergillus* spp., *Penicillium* spp. [5]	Kidney lesions, reduced semen quantity and quality, reduced egg production, reduced body weight, increased kidney weight, alterations in several serum biochemical parameters, immunosuppression [5]	Guidance value	0.25 (feed materials of cereals and cereal products), 0.05 (compound feed for pigs), 0.1 (compound feed for poultry), 0.01 (compound feed for cats and dogs) [15]
T-2, HT-2	*Fusarium* spp. [8]	Reduced body weight, mucosal damage, immunosuppression, infertility of eggs, reduced egg production, reduced feed intake, lesions, serum [8] biochemical parameters, increased heart weight [18]	Guidance value for T-2 + HT-2	0.05 (compound feed for cats) [15]
Zearalenone (ZEN)	*Fusarium* spp. [6]	Increased cervix swelling, increased weight of uterus, liver, ovarian, reduced ovulation rate, reduced fertility [6]	Guidance value	2 (feed materials of cereals and cereal products other than maize by-products), 3 (feed materials of maize by-products), 0.1 (compound feed for piglets, gilts, puppies, kittens, dogs, and cats for reproduction), 0.2 (compound feed for adult dogs and cats other than for reproduction), 0.25 (compound feed for sows and fattening pigs), 0.5 (compound feed for calves, dairy cattle, sheep including lambs, and goats including kids) [15]

^1^ Relative to feed with a 12% moisture content. ^2^ Not available.

## 2. Results

### 2.1. Harvest and Post-Harvest Practices of Cereal Raw Materials

#### 2.1.1. Harvest

The time of harvesting and circumstances during this process affect mycotoxin contamination in the cereals used for the production of feed. Delayed harvesting, for instance, can be a low-cost and effective process by means of maturing and drying cereals at the same time. Nevertheless, this process stresses the crop, in terms of drought, which favors infection and colonization with *Aspergillus flavus* and consequently AF production [19]. Elevated stress levels in the crop also occur with heavy rainfall right before the harvest because the crop is in this case exposed to high moisture after periods of drought. In this situation, delayed harvesting results in an environmental exposure of two extremes and thus in high levels of stress to the crop, which makes the crop more vulnerable to fungal infestation [19]. 

Delayed harvesting elevates the probability of AF production and is also favorable for the production of FUM. This was concluded based on an investigation of maize samples from Brazil that were grown under tropical circumstances. A positive correlation was found between the FUM content in maize and the delay (in days) of the harvest. It was also found that harvesting before the optimal harvest time, even though the crops were mature, resulted in reduced FUM formation [20]. This effect was also reported by Atukwase, et al. [21] and Mansfield, et al. [22]. 

In a study in eastern Turkey, maize was tested for the presence of FUM when maturely harvested at five different time points from September to the beginning of November. The maize was sown at the end of May, and during cultivation, no fungicides or insecticides were applied. Both fumonisin B1 (FB1) and fumonisin B2 (FB2) were detected in the maize samples, and higher mean concentrations were found for FB1 in samples from all time points as compared to FB2. The mean concentration for the total of FB1 and FB2 was the highest, being 6511 µg/kg, in samples harvested at the last time point (5 November 2007). At the first harvest time point (10 September 2007), the mean concentration for the sum of FB1 and FB2 was only 41 µg/kg. At the second time point (24 September 2007), the value was 2856 µg/kg. The mean concentrations of the samples harvested on 8 October 2007 and 22 October 2007 were 1150 and 3099 µg/kg, respectively [23], so a gradual increase over time was seen for the total FB1 and FB2 concentration. Mansfield, Archibald, Jones and Kuldau [22] explained this effect by the fact that more mature maize kernels appeared to have more starch due to more endosperm, which could favor fungal growth and FUM production. Thus, both AF and FUM showed elevated levels in cereal crops under delayed harvested as reported in the literature. 

#### 2.1.2. Drying, Cleaning, and Storage

Post-harvest practices, such as drying, cleaning, and storage, can impact the mycotoxins present in feed materials. 

Cleaning and sorting procedures can lower the mycotoxin and fungal load before storage (to limit the spread of fungal contamination) as well as before processing. The effect of cleaning and sorting of wheat and maize has been investigated in various studies, as recently reviewed by Schaarschmidt, et al. [24,25]. In a study in Tanzania, the AF and FUM contents were three times higher in unsorted maize in comparison to maize that was sorted by removing molded, damaged, and discolored maize kernels. These kernels have higher chances to be contaminated with mycotoxins [26]. A process of continuous cleaning of maize consisting of the steps separating, aspirating, and optical sorting was able to reduce the AF content in the cleaned maize by 65–84% compared to the AF content in the uncleaned maize. The rejected fractions after the maize cleaning process, which was approximately 7% of the uncleaned maize, contained up to 490 µg/kg AF [27]. The impact of cleaning and sorting on DON in raw wheat prior to further processing was studied by Lešnik, et al. [28] and Tibola, et al. [29]. In the study of Lešnik, Vajs, Kramberger, Žerjav, Zemljič, Simončič and Kolmanič [28], wheat was cleaned and sorted at the same time into two classes according to kernel size, 2.0–2.4 mm and >2.4 mm. Further, the difference between samples of wheat cultivated with fungicide treatment and wheat without fungicide treatment was investigated in this research. On average, the cleaning and sorting into smaller kernels did not result in a decrease in the DON content in wheat. The DON concentration in smaller kernels was 1952 µg/kg before cleaning of wheat treated with fungicides and 2024 µg/kg after cleaning. When no fungicides were applied, the difference was larger, with a DON concentration in smaller kernels of 2911 µg/kg before and 3307 µg/kg after cleaning and sorting. In contrast to the smaller kernels, cleaning and sorting seemed to be effective in reducing the concentrations of DON in wheat kernels >2.4 mm. The DON concentration dropped by 23.9% in wheat treated with fungicides and by 27.5% in untreated wheat. In another experiment in this study, decreases in DON concentrations after cleaning and sorting were also observed for the smaller kernels, i.e., 17.3% for fungicide-treated wheat and 11.5% for untreated wheat. In wheat kernels >2.4 mm, concentrations of DON decreased by 39.2% in treated wheat and 54.2% in untreated wheat. The more pronounced reduction in the DON concentrations in the second experiment might be caused by a higher portion of highly contaminated foreign material (such as dust), which was removed by the cleaning procedure [28]. Whereas cleaning and sorting of wheat occurred at the same time in the previous study, these two processes were executed in two steps in the study of Tibola, Fernandes and Guarienti [29]. The cleaning process was executed with the help of an air screen cleaner, and the sorting was done with a gravity separator. Mean concentrations of DON were reduced by up to approximately 3000 µg/kg in samples from different wheat cultivars with cleaning. The consecutive process of sorting at least halved the DON concentrations in the cleaned wheat. The authors concluded that this 2-step cleaning and sorting process largely reduced the DON content as compared to the initial DON content in wheat.

Drying of feed materials is an important process in the production of feed with regards to controlling mycotoxin occurrence and concentrations in feed. Kamala, Kimanya, Haesaert, Tiisekwa, Madege, Degraeve, Cyprian and De Meulenaer [26] investigated drying practices that Tanzanian farmers applied to maize and the effects thereof on the AF and FUM contents in maize. It was noticed that the probability of contamination with these mycotoxins was at least three times higher in maize that was dried on bare ground as compared to maize that was dried on a plastic sheet or on an elevated platform under a veranda. The reasons include the continuous direct exposure of the maize kernels to the soil, which allows moisture to be transferred from soil to maize. Even additional fungal spore transfer is possible when maize is dried on bare ground. In the study of Magembe, Mwatawala and Mamiro [30], storing maize samples on the floor resulted in the highest FB1 concentration in the study, approximately 175 µg/kg, after a 3-month storage period, whereas the FB1 level was approximately 150 µg/kg in maize stored in bags and 125 µg/kg for maize stored in cribs [30]. Solar drying (with a fan and a heater driven by solar panels) was able to reduce the moisture content to 10% after approximately two days, whereas open air drying took between six and 18 days. This also resulted in differences in AF levels in maize. In general, AF concentrations decreased gradually with regards to the moisture content (15%–12%–10%). The highest concentrations of AF were observed in maize stored in polyethylene bags compared to jute or plastic containers [31]. The effect of drum drying on the production of *Penicillium verrucosum* and OTA in rye was studied by Kristensen, et al. [32]. With drum drying, the rye is heated while being transported through a rotating cylinder. A linear decrease in fungal propagules is generally observed when feed materials are dried longer at higher temperatures. Over 70% reduction of active *Penicillium verrucosum* was achieved after drum drying grain at a temperature of 59 °C for 10.5 min. A reduction of 75–100% of *Fusarium* propagules in samples of rye was observed after drum drying at a temperature between 56 °C and 64 °C for 10.5 min. A decrease in fungal propagules was obtained, but not in the OTA content [32]. This shows that the prevention of fungal growth in cereal-based raw materials limits mycotoxins in the final feed product and that this can be achieved by drying. However, drying is not as effective in reducing mycotoxins already present as compared to its limiting effect on fungal activity.

Maize samples were collected both before and after 2-month storage during which the maize was naturally dried. The samples were investigated for the presence of the mycotoxins AFB1, DON, FB1, and ZEN. AFB1 was only detected prior to the storage and the drying process in one sample, at a concentration of 148.4 µg/kg. This could be due to the fact that the water activity (a_w_) of the maize was less than 0.8 after drying, which is not favorable for the growth of *Aspergillus flavus,* resulting in negligible occurrence of AF. Three out of 22 samples were positive for ZEN before as well as after storage and drying. Remarkably, these three samples did not originate from the same sampling site, but were taken from different farms. It is important to note here that the samples before and after storage were not the same samples; they were only collected from the same sampling sites. Amongst the 22 samples before storage and drying, the mean concentration of ZEN was 15.9 µg/kg, while in the samples after storage and drying, it was 85.7 µg/kg. The mycotoxins DON and FB1 were more prevalent than AFB1 and ZEN in this study. DON was present in all samples after storage and drying, and FB1 was detected in all samples. The mean concentration of DON was 80.7 µg/kg in the samples before storage and drying and 1581.2 µg/kg in the samples after storage and drying. For FB1, the mean concentration was 132.8 µg/kg in the samples before storage and drying and 100.2 µg/kg in the samples after storage and drying. The concentrations of FB1 were mostly higher in the samples prior to storage and drying compared to the samples collected after storage and drying. This was not the case for DON; while the concentration of FB1 seemed to be decreased by almost a quarter of the initial concentration, DON increased extensively during the storage and naturally drying process [33].

Proper drying is crucial to avoid *de novo* production of mycotoxins during storage. Neme, et al. [34] observed that there are three parameters that can strongly influence the mycotoxin production during storage: the moisture content of the stored product, temperature, and relative humidity of the storage environment. They reported that *Fusarium* mycotoxins, such as DON and FUM, will not be produced when the a_w_ is less than 0.9. However, a study by Garcia-Cela, et al. [35] showed that maize samples stored at an a_w_ of 0.8 were highly contaminated with AF. In this review, it was reported that the minimum a_w_ for AF production was 0.82, while OTA was still produced at an a_w_ of 0.8. It was concluded that no spoilage would take place provided a_w_ < 0.7. The temperature of the storage environment can play another role in the mycotoxin content of feed materials. The mycotoxin content may decrease as a result of heating, although high temperatures are needed. Temperatures higher than 50 °C can at least prevent mycotoxin production when the activity/survival of fungal propagules is affected. Cool storage of cereals, below 10 °C, can prevent the production of mycotoxins [34]. Although fungi are not eliminated by low temperature, it will minimize fungal growth and metabolism. Production of AF can occur at temperatures from 12 °C to 40 °C. For OTA, this ranges between 10 °C and 25 °C [34].

Garcia-Cela, Kiaitsi, Sulyok, Krska, Medina, Damico and Magan [35] studied three types of maize samples during storage: naturally AF-contaminated maize samples, maize inoculated with *Aspergillus flavus,* and irradiated maize inoculated with *Aspergillus flavus*. These samples were stored under varying conditions of a_w_ (0.8–0.99) and temperature (15 °C–35 °C) for 11 days. In general, more AFB1 was detected than AFB2, and AFG was not detected in any of the samples in this study. The highest contamination of AF occurred in all sample types when stored at a temperature of 30 °C and an a_w_ of 0.95. High AF contamination was also observed in the samples stored at a temperature of 35 °C with an a_w_ of 0.8. A positive correlation was found between dry matter losses during storage and final levels of contamination with AFB1 and AFB2. A study on AFB1, FB1, and FB2 in maize determined the effect of storage with varying conditions, such as temperature (20 °C, 25 °C, and 30 °C) and moisture content (11% and 14%). Although these mycotoxins were present in both maize samples before and after storage, no statistical differences were observed between the different storage treatments [36].

The type of feed (raw) materials, their initial fungal contamination, and the way the materials are stored can impact the mycotoxin content of feed after storage. In the study by Mongkon, et al. [37], maize silage and maize with maize dust were sampled and tested for the presence of AFB1. The maize with maize dust was stored for 30 days. The AFB1 concentration in the maize with maize dust increased by more than 250% as compared to the AFB1 concentration before storage. The maize silage was stored for 14 days. The concentration of AFB1 in silage was doubled after storage compared to the starting concentration. Underlying causes for the large increases can be related to the larger amount of air that had access to these feed materials. In addition, silage has a higher moisture content than other feed materials and has varying storage conditions, which can favor fungal growth and mycotoxin production [37]. 

Maize was sampled and analyzed for FB1 after three, six, and nine months of storage [30]. Although FB1 concentrations after nine months storage were the highest (circa 150–185 µg/kg), differences in FB1 concentrations between samples from different storage periods were insignificant in this study.

Practices that are executed during storage can affect the mycotoxin concentration at the end of the storage period. For instance, maize that was treated with synthetic insecticides during storage was found to have at least five times lower probability to be contaminated with AF as compared to maize without insecticide treatment. For FUM, the probability was halved by the application of insecticides to maize during storage. The authors suggested that the fungal species producing AF (*Aspergillus flavus*) is more affected by the insecticide application to maize and that storage conditions without any measures are more favorable to AF than to FUM [26]. 

In conclusion, conditions during post-harvest practices of feed materials can have an influence on the mycotoxin level therein. The results from the literature study showed that in particular, a high moisture content of the materials, high relative humidity, and a moderate temperature during storage favor fungal growth and mycotoxin production. Cleaning and sorting before storage are important to reduce the fungal load and to limit mycotoxin production during storage. A second cleaning and sorting after storage reduces mycotoxin contamination at the beginning of the feed processing chain.

### 2.2. Processing during Cereal-Based Feed Production

#### 2.2.1. Milling

**Figure 1:**
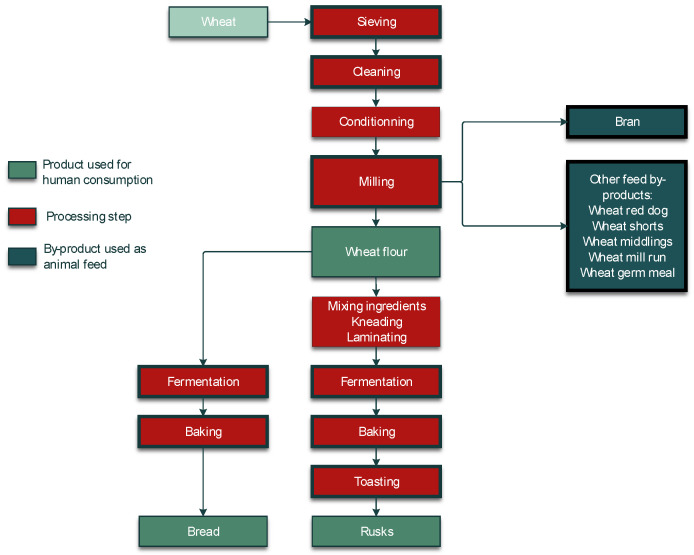
Process diagram of dry-milling of wheat processing into feed and food products.

**Figure 2:**
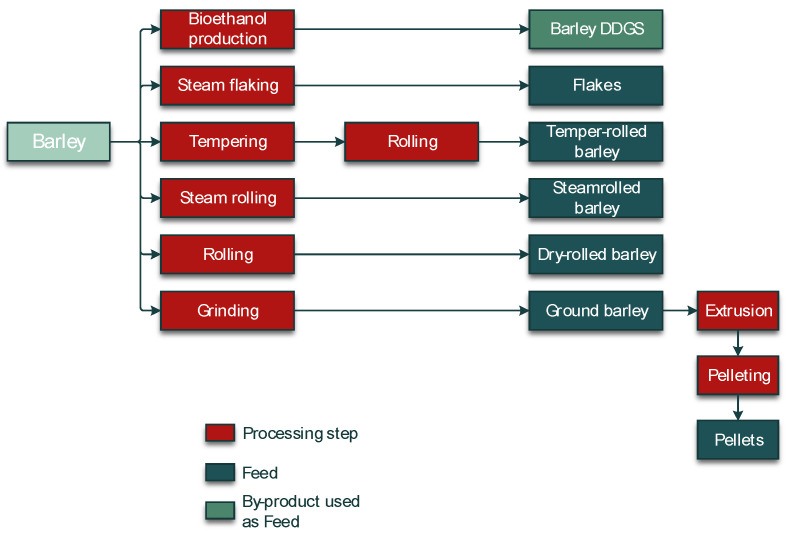
Process diagram of milling and further processing of barley. DGGS: distillers dried grains solubles.

**Figure 3:**
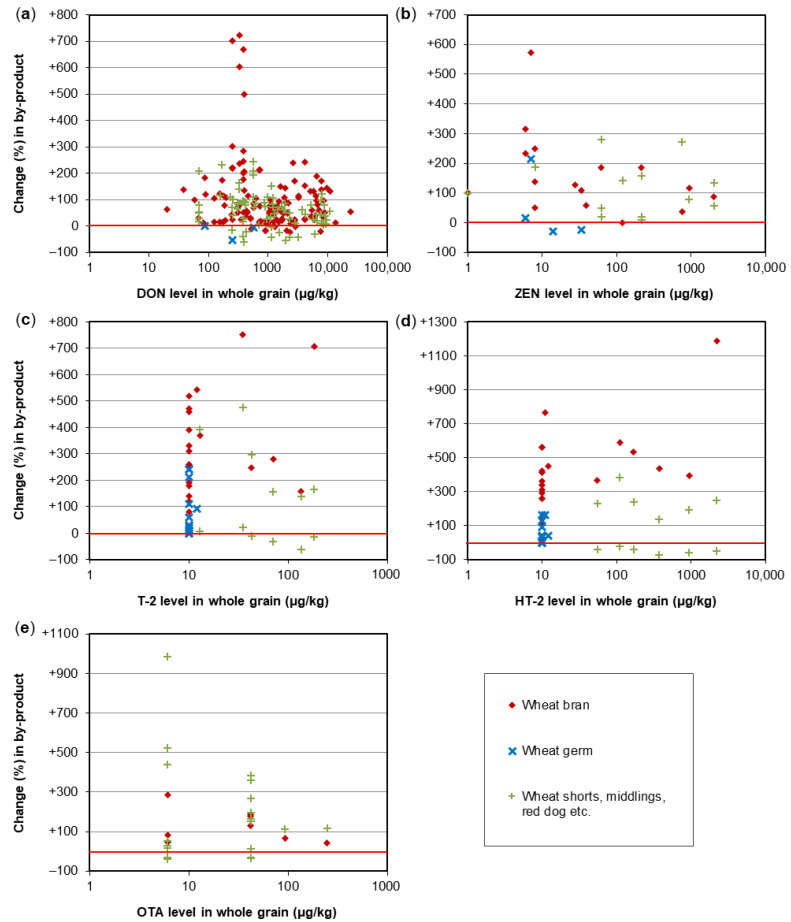
Effect of wheat dry-milling on mycotoxin levels in by-products. (**a**) Deoxynivalenol (DON); (**b**) Zearalenone (ZEN); (**c**) T-2 toxin; (**d**) HT-2 toxin; (**e**) Ochratoxin A (OTA). A change of e.g., +100% represents a 2-fold increase compared to the level in whole grain. The red line indicates no change. Details on the individual studies are provided in Table S1. Information on the dry-milling effect on mycotoxin levels in endosperm fractions of wheat is given elsewhere [24].

**Figure 4:**
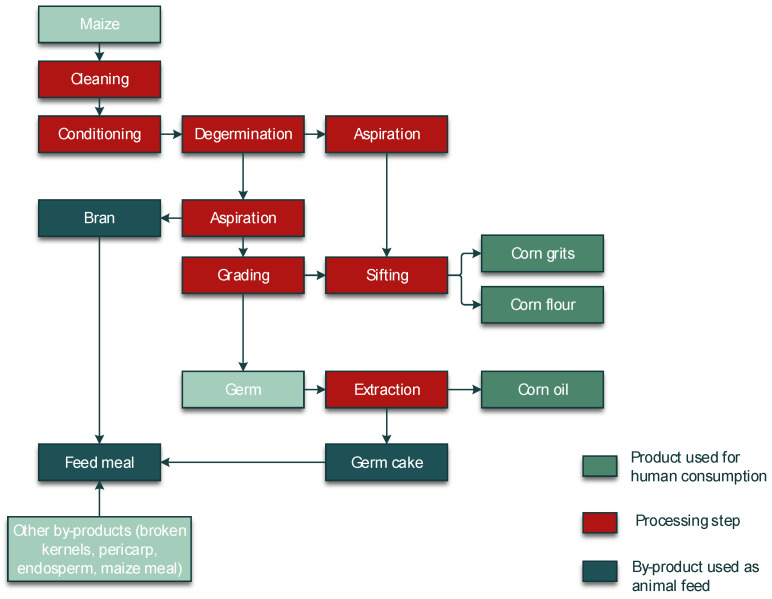
Process diagram of dry-milling of maize.

**Figure 5:**
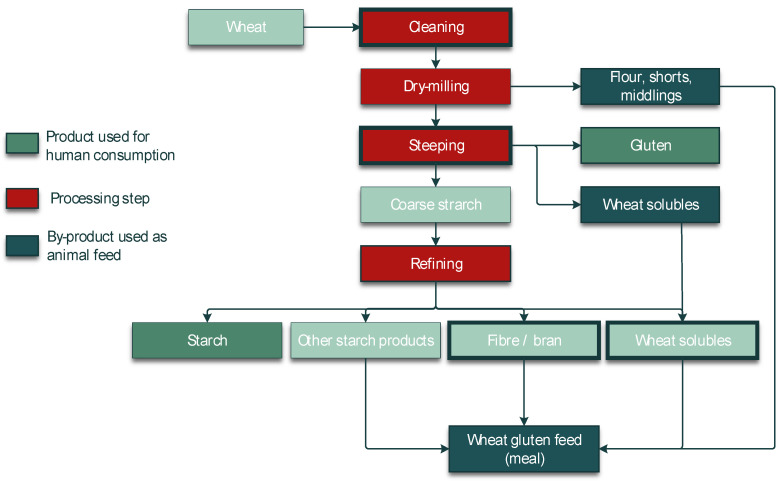
Process diagram of wet-milling of wheat including cleaning and sorting.

**Figure 6:**
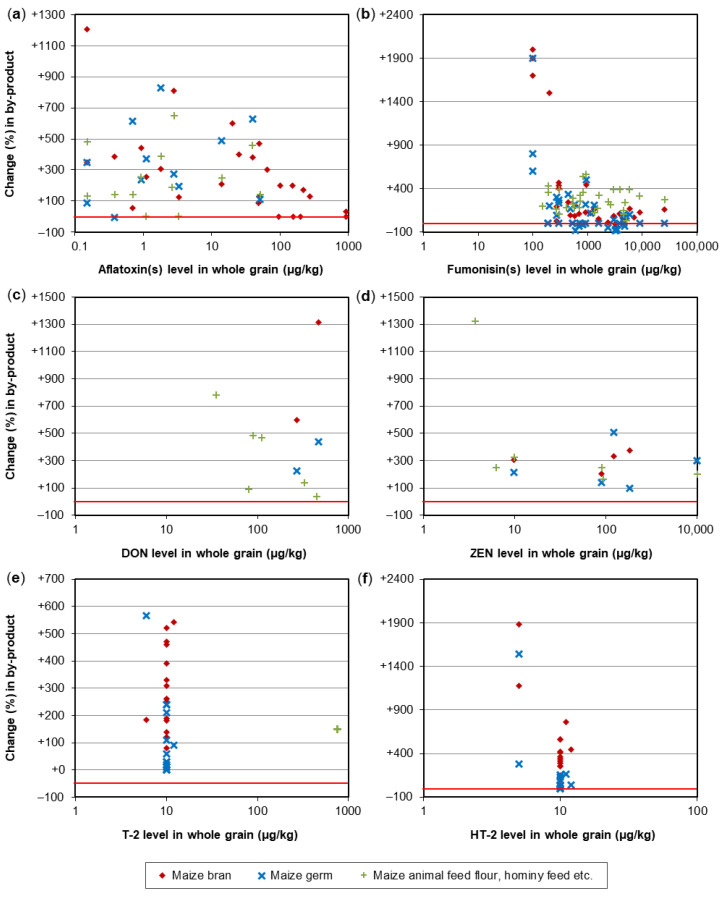
Effect of maize dry-milling on mycotoxin levels in by-products. (**a**) Individual or total aflatoxins; (**b**) Individual or total fumonisins; (**c**) Deoxynivalenol (DON); (**d**) Zearalenone (ZEN); (**e**) T-2 toxin; (**f**) HT-2 toxin. A change of e.g., +100% represents a 2-fold increase compared to the level in whole grain. The red line indicates no change. Details on the individual studies are provided in Table S2. Information about the dry-milling effect on mycotoxin levels in endosperm fractions of maize is given elsewhere [25].

**Figure 7:**
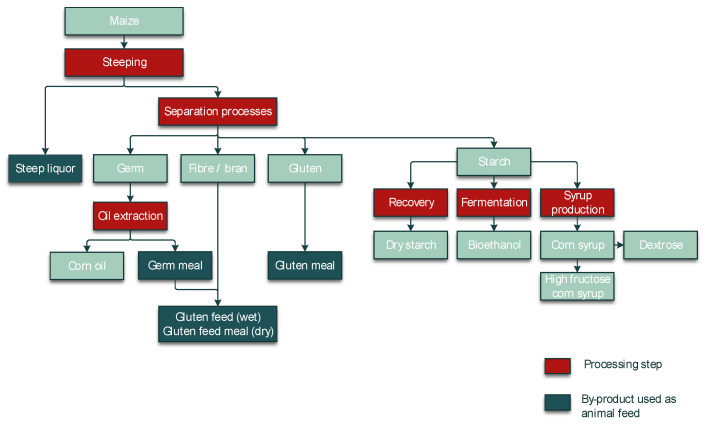
Process diagram of wet-milling of maize.

**Figure 8:**
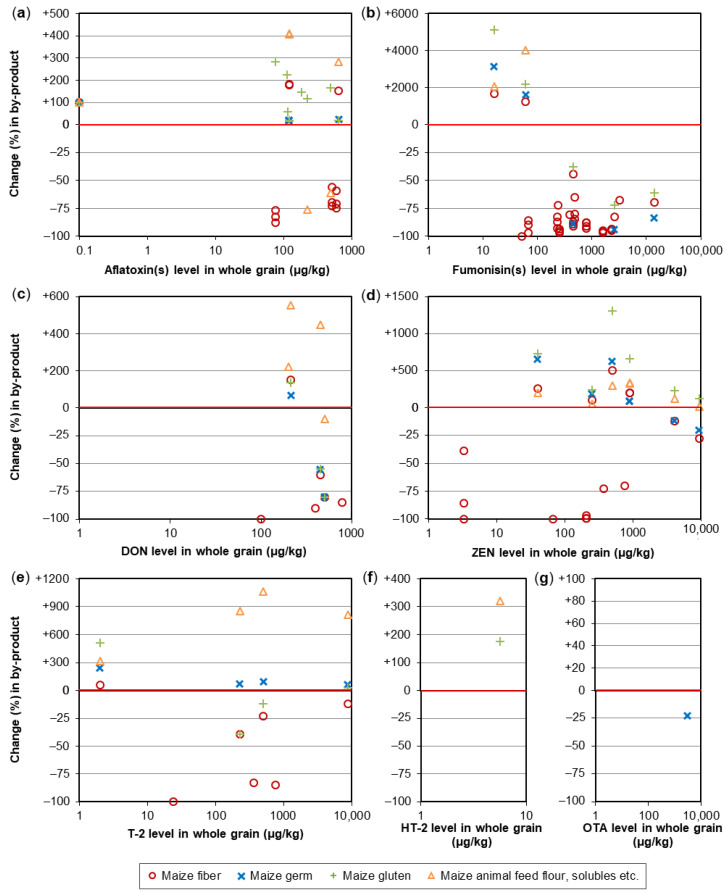
Effect of maize wet-milling on mycotoxin levels in by-products. (**a**) Individual or total aflatoxins; (**b**) Individual or total fumonisins; (**c**) Deoxynivalenol (DON); (**d**) Zearalenone (ZEN); (**e**) T-2 toxin; (**f**) HT-2 toxin; (**g**) Ochratoxin A (OTA). A change of e.g., +100% represents a 2-fold increase compared to the level in whole grain. Please note the different scaling of the *y*-axis in the positive and negative ranges. Details on the individual studies are provided in Table S3. Information about the wet-milling effect on mycotoxin levels in maize starch is given elsewhere [25].

A crucial step in the processing of cereal-based feed is the milling of the kernels. Milling does not eliminate mycotoxins, but it results in redistribution of the amount of mycotoxins into the various milling fractions. These include bran, shorts, break flour, and reduction flour; each of them is used in the further processing of feed. The effects of food processing on mycotoxins in wheat and maize for human consumption were reviewed by Schaarschmidt and Fauhl-Hassek [24,25]. In the current study, we elaborate on the processes relevant to the production of feed. The processes of several types of cereal grain milling, such as dry-milling of wheat and milling of barley, are presented in Figure 1 and Figure 2, respectively. Figure 3 shows the effect of dry milling of wheat on mycotoxin levels in by-products. The process of dry-milling of maize is depicted in Figure 4, and the process of wheat wet-milling is shown in Figure 5. Figure 6 shows the effect of maize dry-milling on mycotoxin levels in maize by-products. The wet-milling process of maize is shown in Figure 7 and the effect thereof on mycotoxin levels in Figure 8. 

By-products of cereal grain dry milling (Figure 1) are typically characterized by higher mycotoxin levels compared to the whole grain. The increase does not seem to depend on the initial concentration in the unfractionated grain (Figure 3 and Figure 6). Many experiments have been performed on the distribution of DON in wheat during milling.

Kushiro [38] observed that the outer parts of the wheat kernel (bran) contained higher levels of DON than inner parts, such as flour after milling. Similar conclusions were drawn by Lee, et al. [39], Lancova, et al. [40], Herrera, et al. [41], Hong, et al. [42], and Belluco, et al. [43]. In these studies, the DON content increased in bran by between 7.3% and 73% relative to the whole grain. Schwake-Anduschus, et al. [44] observed a doubling of the DON content in bran compared to the content in whole grain, Edwards, et al. [45] found a tripling, and the DON concentration in bran was almost five times as high as the initial concentration in the study of Scudamore, et al. [46]. Milling of cleaned grain of wheat varieties named Chikugoizumi and Norin 61 resulted in an increase in DON concentrations of 62% in both the bran and short fractions of the latter wheat variety, whereas in the variety Chikugoizumi, DON increased in shorts but decreased in the bran fraction [47]. Thammawong, et al. [48] reported a decrease of 19.1% of the DON content in bran relative to the whole grain but an increase of 74% in shorts. A reduction of DON in wheat bran of almost 40% compared to the DON content in whole grain by milling was found by Kostelanska, et al. [49]. Pinson-Gadais, et al. [50] ascribed lower production of DON in the bran fraction to biochemical compounds in bran of certain wheat varieties. Overall, for dry-milling by-products of wheat, the levels of DON and ZEN were mostly enhanced by up to 3.5-fold in bran and other bran-containing by-products (Figure 3a,b). Some by-product samples showed lower concentrations, particularly when containing part of the endosperm, such as some middlings. For wheat germ, only limited data are available, indicating mostly no increase in DON or ZEN levels (Figure 3a,b). For example, Giménez, et al. [51] reported a reduction of DON in wheat germ of 53% after dry-milling cleaned whole grain. Levels of T-2 and HT-2 toxins in wheat bran were often increased by up to 6-fold. Wheat germ was found to have higher T-2/HT-2 concentrations than whole grain; but the levels in germ were often lower than those in bran (Figure 3c,d). OTA levels in wheat bran and shorts were approximately 1.5- to 6-fold higher, and some batches of bran flour and shorts flour had lower OTA levels than the whole wheat grain (Figure 3e; Table S1).

Duarte, et al. [52] and Scudamore, et al. [53] found out that OTA is concentrated in the outer layer of cereals, amongst others, wheat. This is supported by the findings of Zebiri, et al. [54]. After cleaning and milling, the OTA concentration in wheat middling was 8.65 µg/kg and in bran, 7.16 µg/kg. The concentrations of OTA in the cleaned wheat was 4.38 µg/kg. Osborne, et al. [55] noticed a difference between the OTA concentrations in milling fractions of soft and hard wheat; whereas milling hard wheat resulted in relatively high concentrations of OTA in bran and offal, these were significantly lower in the milling fractions of soft wheat. The authors reasoned that milling is less effective in soft wheat with the result that milling fractions are not as separated as those of hard wheat.

Milling of cleaned rye seemed to reduce the ergot alkaloids in the milling fraction. The amount of ergot alkaloids in crushed rye was 356 µg/kg and decreased to 239 µg/kg in bran after milling. A greater difference was observed in ergot alkaloids when the peel of rye was removed. A decrease of more than 3000 µg/kg was measured in peeled rye compared to unpeeled rye [56].

A study on barley and the effect of dry-milling focused on the removal of DON by two types of milling. The hulls of the barley kernels were removed through roller milling and precision milling. The roller mill was able to remove 36.7% DON and the precision mill, 85.1% [57]. 

Similar to wheat and barley, mycotoxins in maize are also transferred in high amounts during dry-milling to the bran fraction and other fractions used as feed material. After dry-milling of maize (Figure 4) Schollenberger, et al. [58] showed that the DON concentration was 17 times, the T-2 toxin concentration was almost three times, and the HT-2 toxin concentration almost 13 times higher in bran than it was in the raw maize kernels. High levels of DON were also found in germ meal, screenings, and germ (4056 µg/kg, 2994 µg/kg, and 2543 µg/kg, respectively) [58]. 

FUM increased by 60% in bran compared to raw maize samples after milling in a study of Bryła, et al. [59]. In maize samples that were processed into maize flour for use as feed production, the concentrations of FB1 and FB2 were more than doubled as compared to the unprocessed maize. Concentrations of AFB1, AFB2, AFG1, and AFG2 also showed increases in feed maize flour compared to the unprocessed maize in that study, but the increase was not as high as in the case of FB1 and FB2. The mean concentrations of AFB1 were 91.1 µg/kg in unprocessed maize and 219.6 µg/kg in feed flour. For AFB2, mean concentrations were 3.4 µg/kg and 8.8 µg/kg, respectively. AFG1 showed mean concentrations of 26.3 µg/kg and 48.5 µg/kg and AFG2, of 1.1 µg/kg and 1.7 µg/kg, respectively [60].

The results of the study by Coradi, et al. [61] indicated that smaller particles after dry-milling of maize had higher concentrations of AF and FUM than larger particles. Maize dust, with a density of 0.12 g/cm^3^, had an AF concentration of 166 µg/kg compared to large grain particles with a density of 1.43 g/cm^3^, which had an AF concentration of 18 µg/kg. This was in accordance with the study by Scudamore, et al. [62] in which maize flour with a smaller particle size contained higher concentrations of DON, FB1, FB2, and ZEN than coarse grits. 

In general, for dry milling of maize, most data are available for AF and FUM. Most batches of bran, germ, and other by-products containing bran and/or germ showed elevated levels of AF or FUM—often by up to 6-fold relative to the whole grain (Figure 6a,b). In accordance with the hydrophobic character of FUM, part of the germ samples showed reduced concentrations of FUM. Levels of DON, ZEN, T-2, and HT-2 toxins were elevated in the by-products of maize dry milling, often by 6-fold as compared to whole kernels (Figure 6c–f). Similar to wheat, data for T-2 and HT-2 toxins indicate that levels of these two trichothecenes are somewhat less enhanced in maize germ than in bran (Figure 6e,f). 

Different to dry-milling, wet-milling includes a steeping step that is either applied to flour (typical for wheat; Figure 5) or to whole kernels (in the case of maize; Figure 7). Steeping facilitates separation of the endosperm compounds and can cause translocation of mycotoxins to the liquid fractions, resulting in lower mycotoxin levels in the solid by-products. However, it should be noted that the liquid fractions are often added to the solid by-products to obtain the gluten feed. Magallanes López, et al. [63] compared the effect of dry-milling and wet-milling (both applied to whole grain) on DON in wheat. Whereas dry-milling increased the DON concentrations in bran relative to the concentrations in whole grain, wet-milling was effective in reducing the DON levels in the bran. The DON concentration in the whole grain was 57–61% in the bran of hard red spring wheat and 48–51% in the bran of durum wheat after dry-milling. After wet-milling, the maximum share of DON in bran was 7% and 3% in bran starch from hard red spring wheat and 1% in bran and 5% in bran starch from durum wheat. Most of the DON (97% for hard red spring wheat and 95% for durum wheat) was recovered in the water-soluble fractions, separated from the wet gluten and the starch [63].

Aly [64] studied the distribution of AF in maize during the wet-milling process on a laboratory scale. Milling of maize, after it was steeped in water, resulted in the milling fractions gluten, fibers plus germ, and starch. The starch fraction only contained 16.6%, fiber and 22.4% germ, and gluten contained almost half of the total AF content in the steeped maize (252 µg/kg). The distribution of AFB1, AFB2, AFG1, and AFG2 showed similar patterns between the milling fractions with lower concentrations of AFG1 and AFG2 than AFB1 and AFB2. The steeping step caused high reductions in AFG1 (84.4%) and AFG2 (76%) in the kernels relative to the concentrations before steeping. Another study investigated the effect of wet-milling on AFB1, AFB2, and FB1 in maize. The wet-milling resulted in the fractions endosperm, germ, and pericarp, of which the latter two are used as feed materials. The results showed that the FB1 content in the germ and pericarp fractions as three times higher compared to the maize before wet-milling. The concentrations of AFB1 and AFB2 also increased in these fractions compared to the starting material. The concentrations of AFB1 and AFB2 were 4–6 times higher than the concentrations of these mycotoxins in the starting material [65].

Maize and maize by-products from wet-milling facilities in Korea were studied for mycotoxins in the study of Park, et al. [66]. The unprocessed raw maize samples hardly contained any of the tested mycotoxins: AFB1, AFB2, AFG1, AFG2, OTA, DON, HT-2, T-2, FB1, FB2, and ZEN. AFB1 was detected in low concentrations, with a mean concentration of 0.1 µg/kg, in unprocessed raw maize samples (*n* = 6). In the maize fractions after wet-milling, only AFB1 showed detectable levels (0.2 µg/kg in each fraction and a concentration of 0.3 µg/kg in light steep water and 0.7 µg/kg in maize steep liquor). The latter two fractions are used in the production of feed. OTA showed similar results to those of AF in this study, as it was hardly present in both raw maize samples and samples of wet-milling by-products. Again, the highest mean concentrations of OTA were found in maize gluten (0.5 µg/kg), maize steep liquor (0.8 µg/kg), and light steep water (1.0 µg/kg). Remarkably, OTA was not detected or was only detected in quantities below the limit of quantification in the unprocessed raw maize samples. The highest concentration of DON in unprocessed raw maize was 1385.7 µg/kg. Most of the DON was distributed in the light steep water and maize steep liquor, which contained 82.6% of all DON in the initial maize samples. A reason for this distribution can be due to the fact that DON is highly water-soluble. HT-2 showed a similar pattern as DON, but in lower concentrations with quantities below the limit of quantification. T-2 seemed to be more evenly distributed amongst the milling fractions. The highest quantity of T-2 found in unprocessed raw maize was 10.3 µg/kg. For FB1 and FB2, the highest concentrations in unprocessed kernels were 544.8 µg/kg and 179 µg/kg, respectively. The results for the distribution of FB1 and FB2 among the fractions were more similar to those of DON than of AF. The distribution of FB1 from low to high concentration was starch < bran < germ < gluten < light steep water < maize gluten feed < maize steep liquor. The mean concentration in the latter was 4594.6 µg/kg, 2520 µg/kg in maize gluten feed, and 1585.3 µg/kg in light steep water. For FB2, the distribution in ascending order was starch < light steep water < bran < maize steep liquor < maize gluten feed < germ < gluten. The milling fraction of maize gluten feed had a mean concentration of FB2 of 344.8 µg/kg, germ had a mean concentration of FB2 of 512.2 µg/kg, and gluten had the highest mean concentration of FB2, i.e., 824.9 µg/kg. For ZEN, the highest mean concentrations were observed in corn gluten (329.8 µg/kg), maize germ (298.9 µg/kg), maize bran (142.9 µg/kg), and maize gluten feed (118.2 µg/kg). Initially, the highest concentration of ZEN measured in unprocessed raw maize samples was 200 µg/kg [66]. 

To conclude, wet milling is accompanied by a transfer of part of the mycotoxins to the liquid fractions. Different from wheat, wet milling of maize is applied to the whole kernels and not to the white flour. Thus, maize fiber as by-product of wet milling often has lower mycotoxin concentrations compared to the whole kernels (Figure 8). The reduction might be less pronounced (or absent) in the case of less water-soluble mycotoxins, such as ZEN. The reduction in maize bran is typically accompanied by higher mycotoxin levels in the wet-milling fractions that contain solubles. The concentrated steep liquor is usually added to other wet-milling by-products to form animal feed. Gluten showed, at least in the case of aflatoxins and ZEN, higher contamination levels than the whole grain (Figure 8a,d). In maize germ—which is also used for oil production for human nutrition—ZEN concentrations were mostly elevated, while levels of the highly water-soluble fumonisins were lower in two studies but increased in another. However, data for germ are, in general, relatively rare. In the case of HT-2 and OTA, wet-milling data for maize are overall very rare (Figure 8f,g, Table S3).

Overall, milling may be effective to minimize mycotoxins in products for human consumption, but it may redistribute the mycotoxins into fractions used as feed materials, including liquid fractions in wet-milling. Concentration of mycotoxins in fractions used as feed materials can pose a risk to animal health and of carry-over of mycotoxins in food from animal origin.

#### 2.2.2. Mixing and Mycotoxin Binders

Mixing is a physical treatment of feed; however, it has no effect itself on the mycotoxins present in feed materials. Mycotoxins are not dissipated by mixing, but it may only dilute the level of mycotoxins when contaminated feed materials are mixed with non-contaminated feed materials and may be a side-effect of producing compound feed. Notwithstanding, it is not allowed within the EU to mix inappropriate with appropriate feed materials in order to lower the mycotoxin content in the finished product according to Article 5 of Directive 2002/32/EC [14]. Therefore, the materials of which feed is made should be in compliance with the appropriate regulatory limits. Therefore, the physical removal of mycotoxins is not possible through mixing, and dilution of mycotoxin concentrations in feed is not allowed. Nevertheless, mycotoxin binders can be added during the mixing process. 

Mycotoxin binders do not eliminate the mycotoxins present in feed; instead, they make the mycotoxins less bioavailable to animals. In vitro studies with mycotoxin-contaminated feed showed that the mode of action of mycotoxins bonded by these additives resulted in less severe adverse effects in animals. Examples of mycotoxin binders are active carbon [67] and lactic acid bacteria [68,69]. The latter can be naturally present in the process of ensiling. Since the mycotoxins are not removed physically from feed, the addition of binders does not have an effect on the analytical results of the quantification of mycotoxins in feed in general [70]. For this reason it might be difficult to consider the use of mycotoxin binders in legislation since it would be a challenge to measure the effectivity thereof. Moreover, there is still a lot unknown on the progress and the unbounding of mycotoxins in the animal’s digestive tract. Therefore, more research on the mechanism of these mycotoxin binders is needed.

#### 2.2.3. Extrusion

Thermal processes during feed production may have influence on the mycotoxin content in the finished product. In the study by Ryu, et al. [71], samples of maize grits were spiked with the mycotoxin ZEN, so that the starting material contained 4400 µg/kg ZEN. The experiment with extrusion cooking was conducted with three different temperature parameters, being 120 °C, 140 °C, and 160 °C, with different moisture contents, being 18%, 22%, and 26%. Extrusion was done with two sorts of screws, a mixing screw and a non-mixing screw. In these experiments, the ZEN concentration decreased significantly by between 66% and 83% by extruding the maize grit samples. Concentrations of ZEN were more reduced by extruding the samples with a mixing screw rather than with a non-mixing screw, especially with a lower moisture content. However, a significant relation between the moisture content in the corn grits and the reduction of ZEN was not found. The lowest concentration of ZEN was 730 µg/kg in the sample extruded with the mixing screw at 120 °C and a moisture content of 18%. The sample with the same processing parameters (temperature and moisture content), but extruded with the non-mixture screw, contained 1200 µg/kg after extrusion. Overall, the highest ZEN concentration was 1520 µg/kg in the sample extruded with the non-mixing screw at 160 °C with a moisture content of 22%. For the samples extruded with the mixing screw, the highest ZEN concentration was 1490 µg/kg and was found in the sample extruded at a moisture content of 26% at a temperature of 160 °C [71]. In another study on maize grits, samples were extrusion cooked at two different speeds, 70 and 140 rotations per minute. Further, extrusion took place at temperatures of 150 °C, 175 °C, and 200 °C. In this experiment, reductions of the ZEN concentration were between 66.7% and 80.5%. The highest reduction was achieved when maize was extruded at 150 °C and at a speed of 140 rotations per minute. The lowest reduction in the ZEN content in maize was observed at an extrusion temperature of 200 °C and a speed of 70 rotations per minute, [72].

Samples of wholemeal wheat were investigated for DON and ZEN after extrusion cooking at temperatures of 140 °C, 160 °C, and 180 °C. For DON, most of the samples showed a reduction after extrusion. Only in samples extruded at 160 °C with higher moisture contents (21% and 17%) and at 180 °C with a 17% moisture content did the DON level not decrease. The highest decrease in DON was 23.4% in the sample extruded at 160 °C with a 15% moisture content. At all temperatures, the levels of DON in samples with a 15% moisture content were reduced the most. At temperatures of 140 °C and 180 °C with a moisture content of 15%, DON was decreased by 18.9% and 20.5%, respectively. Whereas extrusion was able to reduce the DON concentration in wheat samples under certain circumstances, the ZEN level was hardly reduced. A significant reduction of the ZEN level (17.4%) was only observed at a temperature of 140 °C in the samples extruded at a moisture content of 21%. Instead, in almost all samples, ZEN increased between 2.5% and 15.4% during the extrusion of wholemeal wheat. Remarkably, the highest increase was in the sample at 140 °C with a low moisture content (15%). The authors concluded that the moisture content had a stronger influence on the mycotoxin content than temperature [73].

#### 2.2.4. Ensiling

Despite high-moisture conditions, the process of ensiling exerts conditions that are less optimal for fungal growth. For this reason, ensiling can have an impact on the production of mycotoxin in feed materials. While the temperature during ensiling can still fall within the temperature range of fungal growth (10–40 °C), pH during ensiling is not optimal for the growth of fungi. Fungal growth is possible between a pH of 4 and 8. The pH in silage may drop to around 4, which may slow fungal growth. Although factors may differ per species, the lack of oxygen under the anaerobic ensiling conditions makes the ensiling environment in general less favorable for fungal growth and also for mycotoxin production [74].

In the study of Sultana, et al. [75], samples of green maize fodder were ensiled anaerobically in a laboratory set-up with the addition of the bacteria *Enterococcus faecium* and *Lactobacillus plantarum*. Neither AFG1 nor AFG2 were detected in either fresh or ensiled samples. The mean concentration of AFB1 was slightly lower after ensiling as compared to before, but the mycotoxin was more prevalent in ensiled samples with an increase of 4.2%. Exposure of silage to oxygen when feed is taken out can cause an increase in mycotoxin production. OTA in this study a higher prevalence in fresh maize fodder (54.2%) as compared to the ensiled samples (20.9%). Not only did the prevalence of OTA decreased, but the mean concentration of the contaminated samples decreased from 8.03 µg/kg to 3.98 µg/kg [75]. The reduction in mycotoxins in ensiled samples can be caused by the bacterial activity in the silage, as some bacteria may bind or degrade mycotoxins.

Wang, et al. [76] investigated the presence of several mycotoxins in raw and ensiled maize kernels at a laboratory scale. The latter samples were ensiled for 55 days at different temperatures, namely 20 °C, 28 °C, and 37 °C, in polyethylene jars with a volume of 1 L. The silages were treated with the bacteria *Lactobacillus plantarum* and *Pediococcus pentosaceus,* and one silo with no addition functioned as a control. After ensiling, the highest AFB1 concentration was 24.5 µg/kg in the control silo with a temperature of 20 °C. The AFB1 concentration before ensiling was 30.26 µg/kg, so a slight decrease was observed during the ensiling process. This was not the case for OTA, for which the concentrations in the ensiled samples were all higher, even without the addition of bacteria, than in the sample before ensiling, being 34.45 µg/kg. In contrast to AFB1, for OTA it seemed that silage at a higher temperature resulted in higher OTA concentrations. The highest OTA concentration was 51.6 µg/kg and was measured in the control silo at a temperature of 37 °C. The samples with the added bacteria showed lower concentrations, but the OTA content also increased under higher temperature. Ensiling of maize samples significantly decreased DON. The mean concentration of the samples before ensiling was 163.2 µg/kg, and the mean concentrations of ensiled samples without addition were between 38.3 µg/kg and 56.4 µg/kg (depending on the ensiling temperature). Nevertheless, the silage with addition showed higher values for DON, being 179 µg/kg for *Lactobacillus plantarum* at a temperature of 37 °C and 261 µg/kg for *Pediococcus pentosace* at a temperature of 20 °C. DON was more concentrated in the top section of the silo. No significant changes were observed in the concentrations of T-2, HT-2, and ZEN before and after ensiling [76]. 

Cavallarin, et al. [77] also measured higher mycotoxin concentrations in silage from the top section of the silo, AF to be specific. According to these authors [77], this could be explained by the fact that the silage in the top section in the silo had higher chances of being exposed to oxygen than the other sections. Oxygen exposure would result in fungal growth. The same concerns were shared by Ferrero, et al. [78], who warned that although the environmental conditions during ensiling may stop fungal growth and mycotoxin production, fungi may survive. The results of their study showed that *Aspergillus flavus* revived and started producing AF when the silage was exposed to oxygen after the ensiling period. Then, the amount of AF increased significantly. Nevertheless, the increases were smaller in silages in which the bacterium *Lactobacillus buchneri* was added and even less with the addition of a mixture of the bacteria *Lactobacillus buchneri* and *Lactobacillus hilgardii* [78]. 

Keller, et al. [79] monitored the mycotoxins AFB1, AFB2, AFG1, AFG2, and OTA in 464 maize samples before and after they were ensiled. The mycotoxins AFB1, AFB2, and OTA were all detected in maize before ensiling and after ensiling, whereas none of the samples showed detectable levels of AFG1 or AFG2. Increases in AFB1 and AFB2 concentrations were found in ensiled maize samples compared to the raw maize samples. This was not the same for OTA, where no significant difference was observed in the concentrations before and after ensiling. The samples were taken from several sections within the silos: the central part, the laterals, the lower layer, and the upper layer. However, in this study, the mycotoxins seemed to be distributed evenly throughout these sections [79]. An increase in mycotoxin concentration of maize after ensiling was also observed for DON. The mean concentration in maize before ensiling was 39 μg/kg and 179 μg/kg after 60 days of ensiling. After the 60th day, a small reduction in the DON concentration was observed. The DON concentration was 153.9 μg/kg after 100 days of ensiling [80]. Similar results were obtained in the study by Jensen et al. (2020). The DON concentration in maize samples increased from 2096 µg/kg to 2805 µg/kg after 90 days of ensiling. However, the process of ensiling did not have an effect on the concentrations of ZEN in maize [81].

Pereyra, et al. [82] investigated the mycotoxins AFB1, DON, FB1, and ZEN in samples of whole-plant maize before and after it was fermented in trench-type silos. The authors noticed that mycotoxin concentrations increased during the ensiling process, except for ZEN, for which no significant differences were observed. The mean concentrations of DON and FB1 increased from 150 µg/kg to 276 µg/kg and from 600 µg/kg to 1110 µg/kg, respectively. Remarkably, none of the samples before ensiling contained detectable levels of AFB1, but this toxin was found in the samples after ensiling. Although the authors explain that the upper, lower, and border sections of the trench-type silo featured pH conditions that were favorable for fungal production (pH > 6), in this study, only samples from the middle section contained AFB1 after ensiling (6 out of a total of 90 samples). The concentrations of AFB1-positive samples ranged from 1.43 µg/kg to 155.78 µg/kg [82]. 

Potential degradation and/or binding of mycotoxins by bacteria during ensiling have been experimented with and described. Mansfield, et al. [83] measured samples of freshly cut maize and maize silage for DON. The occurrence of DON as well as the concentration of DON was lower in maize after it was ensiled. The freshly cut maize tested positive for DON in 75% of the samples, while this was 42% of the silage samples. Ensiling of the maize samples decreased the average DON concentration from 1400 to 600 µg/kg. In another study by Mansfield, Archibald, Jones and Kuldau [22], samples of maize silage were analyzed for FB1 and FB2. In contrast to their hypothesis on DON from the previous study, the results of this study did not support that degradation or binding of FB1 and FB2 took place in the maize samples (*n* = 20) during ensiling.

Besides fermentation during ensiling, the process of fermentation was examined in a study with maize meal. Samples (*n* = 8) of maize meal were spiked with the mycotoxins FB1 and ZEN in quantities of 1970 µg/kg and 1850 µg/kg, respectively. The difference in the fermentation of the meal was studied between samples with lactic acid bacteria (LAB) cultures and without the addition of LAB cultures. In the samples with LAB added, a reduction of 74.6% in FB1 concentration was achieved. For ZEN, this was a maximum of 68.2%. In the samples without the addition of LAB, the highest reduction achieved in FB1 concentration was 24.4% and for ZEN, 34.3%. A linear relationship between the fermentation time and decrease in mycotoxin concentrations was observed. The authors concluded that LAB cultures could be applied to reduce FB1 and ZEN levels [84]. In a monitoring study, which analyzed the mycotoxin content in maize before and after ensiling, degradation of mycotoxin was observed. However, only the difference observed for the concentrations of ZEN in maize and maize silage was significant. For the other mycotoxins, being DON, FB1, FB2, and FB3, no significant difference was observed between the concentrations in maize and maize silage [85].

In general, binding and/or degradation of mycotoxins might occur by bacterial activity during the process of ensiling. Moreover, environmental conditions during ensiling become less favorable for *de novo* mycotoxin production. Nonetheless, several studies reported increased mycotoxin contents in silage, especially in the top section of the silo. This might be due to the less anaerobic environment than the rest of the silo, which provides an opportunity for fungi to produce mycotoxins.

## 3. Conclusions

Due to their adverse effects on humans and animals upon ingestion, the presence of mycotoxins in food and feed should be avoided. The production processes of cereal-based feed materials and the influence on the mycotoxin content therein are presented in this review. The time of harvest of cereals can influence the mycotoxin content. Multiple findings show that a delayed harvest resulted in higher mycotoxin concentrations. It was even seen that harvesting before the optimal maturity of the cereal would result in a lower mycotoxin content. Post-harvest practices, such as drying, fungicide application before storing, and cleaning and sorting impact the development of mycotoxins in feed material during storage. Whereas the application of fungicides and cleaning and sorting before storing of the raw materials lowered the presence of DON in wheat, the occurrence and concentrations of DON and ZEN were higher after natural drying and those of AFB1 after storage. For processing, milling and the effects thereof on mycotoxins are abundantly described in the literature. Overall, mycotoxins were mostly present in the outer layers of the grain kernel. Thus mycotoxins were not removed, but were more concentrated in the bran-containing fractions after milling. Whereas mycotoxins were concentrated in these fractions after dry-milling, wet-milling minimized the presence of mycotoxins even in the fiber fraction and were distributed to the liquid fractions. However, liquid by-products from wet-milling can also be used in the production of feed. Mixing of raw materials did not eliminate mycotoxins. Nevertheless, one can add feed additives during this process that bind mycotoxins. In this way, the ingestion of mycotoxins will exert adverse effects in the animals to lesser extent; however, the use of mycotoxin binders requires a change in legislation due to the fact that mycotoxins are still measured in feed. Moreover, there is still a possibility that mycotoxins can reach the animal’s digestive tract in an unbound state, thus still exerting adverse effects on the animal. The effect that extrusion can have is variable. This can be described to the multiple variables of this process, such as the moisture content, temperature, screw type, screw size, and speed. Some studies suggested that the moisture content and temperature could play a role in reducing mycotoxins. For example, some studies showed that a moisture content of 20% had higher reductions (e.g., for OTA); however, another showed that a moisture content of 15% exerted the highest reduction effect on DON. For temperature, the highest reductions in DON and OTA were obtained after extrusion at temperatures between 160 °C and 180 °C, but the highest reduction in ZEN was at a temperature of 120 °C. Overall, the process of extrusion was able to reduce the mycotoxin content. In addition to pelleted feed materials, feed materials can also be ensiled. The circumstances during this process have an influence on the mycotoxin content. Results differed between studies on the effect of ensiling on mycotoxins. However, several studies identified that access to oxygen could be a supporting factor in the production of mycotoxins during this process.

Although fungi, and as a result, mycotoxins are naturally present in cereal-based raw materials, efforts should be taken to minimize the presence thereof in food and feed due to the adverse effects on humans and animals. For this reason, it is important that factors and processes that influence the mycotoxin content are studied and identified. Understanding the effects of processes during the production of feed on mycotoxins could be of great help in mitigating the risk of mycotoxins in animals and, consequently, in humans. Further research should be conducted on influential factors with contradictory results, e.g., ensiling. For the innovative influential factors, such as the use of mycotoxin binders, more research should focus on their effectivity.

## 4. Materials and Methods 

A systematic literature review was performed using four different databases: Scopus, Web of Science, Biological Abstracts, and CAB Abstracts, with references published in English language during the period between 2000 and March 2022. The search focused on the mycotoxins for which guidance values and maximum limits for animal feed have been established. Only research articles were included (excluding review papers) reporting on studies in which an instrumental analytical method was used for mycotoxin analyses, excluding studies that used immuno-based methods. Search strings were defined beforehand, and were as follows:

#1 Feed

feed OR “feeding stuff” OR “animal food” OR wheat OR triticum OR barley OR “Hordeum vulgare” OR maize OR corn OR “Zea mays” OR bran OR germ OR grain* OR cereal* OR silage

AND

#2 Hazard

mycotoxin* OR aflatoxin* OR AF OR deoxynivalenol OR DON OR fumonisin* OR FUM OR “ochratoxin A” OR OTA OR “T-2” OR “HT-2” OR zearalenone OR ZEN OR “rye ergot”

AND

#3 Processing factors

“processing factor*” OR “effect* of processing” OR “processing effect*” OR “influence* of processing” OR “processing influence*” OR “effect* of handling” OR “influence of handling” OR “handling influence*” OR fermentation OR ferment* OR saccharification OR centrifugation OR rolling OR steaming OR mixing OR drying OR grinding OR distillation OR milling OR cleaning OR storage* OR transport*

AND

#4 Method of analysis

“LC-MS” OR “liquid chromatography-mass spectrometry” OR “HPLC” OR “High-performance liquid chromatography” OR “UPLC” OR “ultra-performance liquid chromatography” OR “UHPLC” OR “ultra-high-performance liquid chromatography” OR “GC” OR “gas chromatography” OR “GC-MS” OR “gas chromatography-mass spectrometry”

AND NOT

#5 Food commodities (exclusion terms)

coffee OR beverage* OR wine OR milk OR dairy OR chilli* OR bread OR fruit OR tempeh 

The search strings #1, #2, and #3 were performed to appear in the title, abstract, and keywords in the articles. The appearance of search string #4 was not limited to a specific section in the articles since methods of analysis are often described in the Methods and Materials section of a paper. The search string #5 was only inserted to exclude these articles when the search string appeared in the title. After importing all the references retrieved through the respective four databases into an Endnote file, the duplicates were removed. After deletion of duplicates, 989 references remained. A division was made between relevant hits, not relevant, and maybe relevant references, on the basis of scanning the title and abstract of each hit. During this selection, a reference was found relevant when the respective study concerned one or more influential factors regarding mycotoxins in cereal raw materials used for feed production or influential factors during cereal-based feed production. References were found not relevant when the particular study described processes of food production that were not applicable to feed processing. Even when a paper was published before 2000 and thus was outside the time span of the search, it was still selected as relevant when the abstract showed that the paper may be in the scope of this review. From these, 183 articles were found to be relevant for the scope of this research. The full text was read from the relevant papers, and data on the processing effects were extracted into an Excel file. In cases where the articles were deemed not relevant after reading the full text, the papers were not included in the review.

An additional search was conducted for literature on dry- and wet-milling effects on mycotoxin concentrations in milling by-products used as feed materials. This literature search also made use of the snowball method and was not limited to a defined time span, but covered literature published before April 2020. The references considered are presented in the Supplementary Material.

## Supplementary Materials

The following are available online at https://www.mdpi.com/article/10.3390/toxins14050301/s1, Table S1: Effect of wheat dry milling on mycotoxin concentrations in by-products [29,39,40,43,47,48,49,51,53,86,87,88,89,90,91,92,93,94,95,96,97,98,99,100,101,102,103,104,105,106,107,108,109], Table S2: Effect of maize dry milling on mycotoxin concentrations in by-products [58,59,60,108,110,111,112,113,114,115,116,117,118,119,120,121,122,123,124,125,126,127], Table S3: Effect of maize wet milling on mycotoxin concentrations in by-products [64,66,114,128,129,130,131,132,133,134,135,136,137], and list of references cited in the supplementary tables.
