# Supplementary material for: Factors during Production of Cereal-Derived Feed That Influence Mycotoxin Contents
Source: Toxins (Basel). 2022 Apr 25;14(5):301. doi: 10.3390/toxins14050301 (PMC9143035; doi:10.3390/toxins14050301)
Supplement: Supplementary file 1 [file toxins-14-00301-s001.zip › toxins-1660313-supplementary.pdf]

Supplementary Materials: Factors during Production of Cereal-Derived Feed That Influence Mycotoxin Contents

Yvette Hoffmans, Sara Schaarschmidt, Carsten Fauhl-Hassek and H.J. van der Fels-Klerx

Table S1. Effect of wheat dry milling on mycotoxin concentrations in by-products.

Milling procedure/ technology [a]	Type of wheat and variety	Origin	Type of cultivation	Harvest year	Type of contamination	Myco-toxin	Initial level in whole grains (µg/kg)	Change [%] [b] – bran	Change [%] [b] – germ	Change [%] [b] – other by-products (shorts, middlings, red dog etc.)	Comments	Refer-ence
Experimental milling in a semi-industrial semolina mill (roller mill)	Durum wheat	Italy (Northern)	(not specified)	(not specified)	natural	DON	250	Bran: ~ +220	–	Fine middlings: ~ -16	–	[1]
	Durum wheat	Italy (Northern)	(not specified)	(not specified)	natural		430	Bran: ~ +56	–	Fine middlings: ~ -23	–	
	Durum wheat	Italy (Northern)	(not specified)	(not specified)	natural		700	Bran: ~ +16	–	Fine middlings: ~ +6	–	
	Durum wheat, variety Bracco	Italy (Northern)	experimental (incl. pesticide treatment)	2002	inoculation in the field with *Fusarium culmorum* and *Fusarium graminearum*		1,530	Bran: ~ +92	–	Fine middlings: ~ -24	–	
	Durum wheat, variety Orobel	Italy (Northern)	experimental (incl. pesticide treatment)	2002	inoculation in the field with *Fusarium culmorum* and *Fusarium graminearum*		1,970	Bran: ~ +89	–	Fine middlings: ~ -56	–	
	Durum wheat, variety Orobel	Italy (Northern)	experimental (without pesticide treatment)	2002	inoculation in the field with *Fusarium culmorum* and *Fusarium graminearum*		3,220	Bran: ~ +113	–	Fine middlings: ~ -42	–	

	Durum wheat, variety Bracco	Italy (Northern)	experimental (without pesticide treatment)	2002	inoculation in the field with *Fusarium culmorum* and *Fusarium graminearum*		6,130	Bran: ~ +39	–	Fine middlings: ~ -30	–	
	Durum wheat, variety Duilio	Italy (Northern)	experimental (incl. pesticide treatment)	2002	inoculation in the field with *Fusarium culmorum* and *Fusarium graminearum*		6,630	Bran: ~ +189	–	Fine middlings: ~ +21	–	
	Durum wheat, variety Duilio	Italy (Northern)	experimental (without pesticide treatment)	2002	inoculation in the field with *Fusarium culmorum* and *Fusarium graminearum*		9,710	Bran: ~ +142	–	Fine middlings: ~ +22	–	
Experimental milling in a semi-industrial semolina mill (roller mill)	Durum wheat, variety Acalou	France	(not specified)	2003	natural	**DON**	382	Sized fine bran: +51<br>Purified fine bran: +200<br>Coarse bran: +283	–	Shorts: +18	–	[2]
	Durum wheat, variety Acalou	France	(not specified)	2003	natural		4,203	Sized fine bran: +24<br>Purified fine bran: +153<br>Coarse bran: +243	–	Shorts: +45	–	
Experimental milling in a Chopin CD1 mill	Durum wheat	Spain	(not specified)	(not specified)	natural	**DON**	24,100	Bran: +53	–	–	–	[3]
Industrial milling using a conventional hammer mill	30 batches of durum wheat (blends of grains stocks from two to five different geographic origins)	France, Greece, Iran, Italy, Mexico, Syria, Turkey, USA	commercial (not further specified)	(not specified)	natural	**DON**	mean: 69 (range: n.d.–272)	–	–	Shorts (middlings): mean: +52<br>Flour shorts: mean: +18	Individual data were not provided in the study	[4]

Industrial milling using a hammer mill with optical sorter and debranner	30 batches of durum wheat (blends of grains stocks from two to five different geographic origins)	France, Greece, Iran, Italy, Mexico, Syria, Turkey, USA	commercial (not further specified)	(not specified)	natural	**DON**	mean: 69 (range: n.d.–272)	–	–	Shorts (middlings): mean: +208<br>Flour shorts: mean: +78	Individual data were not provided in the study	
Industrial milling incl. debranning	different batches of durum wheat	(not specified)	commercial (not further specified)	(not specified)	natural	**DON**	382	Bran layer 1 (outer): ~ +670<br>Bran layer 2 (middle): ~ +244<br>Bran layer 3 (inner): ~ +175	–	Bran shorts: ~ +95<br>Flour middlings: ~ -40	–	[5]
							393	Bran layer 1: ~ +498<br>Bran layer 2: ~ +206<br>Bran layer 3: ~ +135	–	Bran shorts: ~ +28<br>Flour middlings: ~ -61	–	
							252	Bran layer 1: ~ +702<br>Bran layer 2: ~ +302<br>Bran layer 3: ~ +218	–	Bran shorts: ~ +101<br>Flour middlings: ~ +42	–	
							328	Bran layer 1: ~ +723<br>Bran layer 2: ~ +604<br>Bran layer 3: ~ +235	–	Bran shorts: ~ +164<br>Flour middlings: ~ +140	–	
Industrial milling using 75%-sorted milling	Different batches of hard (durum) wheat	Russia	commercial (not further specified)	1986-1992	natural	**DON**	500	Bran: +29	–	–	–	[6]
							1,200	Bran: +7	–	–	–	
							1,200	Bran: +78	–	–	–	
							1,600	Bran: +57	–	–	–	
							2,100	Bran: +47	–	–	–	
Experimental milling in a modified laboratory mill (LM 80042) combined with a laboratory sifter and air purifier	Different batches of hard (durum) wheat	Russia	(not specified)	1986-1992	natural	**DON**	1,080	Bran: +100	–	–	–	
							2,160	Bran: +88	–	–	–	

Experimental 75%-sorted milling in a laboratory Nagema mill	Soft wheat	Russia	(not specified)	1986-1992	natural	**DON**	720	Bran: +211	–	–	–	
Experimental one-sort milling in a laboratory Bühler mill (MLU)	Different batches of soft wheat	Russia	(not specified)	1986-1992	natural	**DON**	2,600	Bran: +240	–	–	–	
							2,800	Bran: +169	–	–	–	
							700	Bran: +211	–	–	–	
							5,400	Bran: +61	–	–	–	
							6,600	Bran: +96	–	–	–	
							5,100	Bran: +133	–	–	–	
							5,800	Bran: +132	–	–	–	
							3,200	Bran: +107	–	–	–	
							1,100	Bran: +78	–	–	–	
							1,300	Bran: +61	–	–	–	
Industrial milling in an Argentinean mill	Batch (with 24x6 subsamples) of wheat *(not further specified)*	(not specified)	commercial (not further specified)	2001	natural	**DON**	mean: 1928 (range: 609–3,345)	Bran: mean: ~ +143	–	–	–	[7]
Industrial milling in UK mills	35 batches of wheat *(not further specified)*	(not specified)	commercial (not further specified)	2004-2007	natural	**DON**	mean: 87 (range: 16–481)	Bran: mean: +182	Germ: mean: ca. 0	–	Individual data were not provided in the study	[8]
	Different batches of wheat *(not further specified)*	(not specified)	commercial (not further specified)	2004-2007	natural	**ZEN**	6	Bran: ~ +233	–	–	–	
							8	Bran: ~ +50	–	–	–	
							7	Bran: ~ +571	Germ: ~ +214	–	–	
							6	Bran: ~ +317	Germ: ~ +17	–	–	
							8	Bran: ~ +250	–	–	–	
							34	Bran: ~ +109	Germ: ~ -24	–	–	

Experimental (pilot-scale) milling in a Bühler mill (MLU-202)	10 batches of 5 wheat varieties (Claire, Malacca, Nijinsky, Robigus, Xi19)	(not specified)	(not specified)	2004	(not specified)	**DON**	mean: ~2,308 (range: ~180–14,000)	Bran: mean: -23	–	Shorts (offal): mean: ca.+50<br>Bran finisher flour: mean: ca. -44<br>Shorts (offal) finisher flour: mean: +26	<u>Overall</u>: Individual data were not provided in the study; circa values are taken from diagrams	
	10 batches of 7 wheat varieties (Claire, Deben Hereward, Malacca, Nijinsky, Paragon, Xi19)	(not specified)	(not specified)	2005	(not specified)		mean: ~1,129 (range: ~230–5,300)	Bran: mean: +67	–	Shorts (offal): mean: ca. +150<br>Bran finisher flour: mean: ca. -44<br>Shorts (offal) finisher flour: mean: -4		
	10 batches of 5 wheat varieties (Claire, Malacca, Nijinsky, Robigus, Xi19)	(not specified)	(not specified)	2004	(not specified)	**ZEN**	mean: ~215 (range: ~37–720)	Bran: mean: ca. +185	–	Shorts (offal): mean: +157<br>Bran finisher flour: mean: ca. +20<br>Shorts (offal) finisher flour: mean: ca. +10		
	10 batches of 7 wheat varieties (Claire, Deben Hereward, Malacca, Nijinsky, Paragon, Xi19)	(not specified)	(not specified)	2005	(not specified)		mean: ~62 (range: ~ <5–470)	Bran: mean: ca. +185	–	Shorts (offal): mean: +280<br>Bran finisher flour: mean: ca. +20<br>Shorts (offal) finisher flour: mean: +50		
Industrial milling in a Spanish mill	36 batches of wheat *(not further specified)*	Spain, France, Germany, USA	commercial (not further specified)	2009, 2010	natural	**DON**	mean: 251 (range: >LOD to 820)	–	Germ: mean: -53	–	Individual data were not provided in the study	[9]
	Wheat *(not further specified)*	(not specified)	commercial (not further specified)	2009 or 2010	natural	**ZEN**	14	–	Germ: -29	–	–	
Industrial milling in a Canadian mill	Ontario soft white winter wheat (mixture of 2 batches)	Canada	commercial (not further specified)	1982	natural	**DON**	420	Bran: +37	–	Shorts: ~ +80<br>Middlings: +87	–	[10]

Experimental milling in a pilot mill	Ontario soft white winter wheat	Canada	(not specified)	(not specified)	natural	**DON**	620	Bran: ~ +105	–	Shorts: ~ +85	–	
Experimental milling in a Bühler laboratory mill	Ontario soft white winter wheat	Canada	(not specified)	(not specified)	natural	**DON**	580	Bran: ~ +70	–	Shorts: ~ +70	–	
Experimental milling in a Bühler laboratory mill	Different batches of Quebec hard red spring wheat	Canada	(not specified)	(not specified)	natural	**DON**	8,660	Bran: +30	–	Shorts: +78	–	
							970	Bran: +21	–	Shorts: +108	–	
Experimental milling in a pilot mill	Batch of hard red spring wheat, variety Concord	Canada	commercial (not further specified)	1981	natural	**DON**	1,190	Bran: ~ +13	–	Shorts: ~ +69	–	[11]
	(Commercial) batch composed of different varieties of hard red spring wheat	Canada	commercial (not further specified)	1981	natural		7,610	Bran: ~ -22	–	Shorts: ~ +45	–	
Experimental milling of scoured grain in a Bühler pilot mill (small commercial-scale mill)	Batch of hard red spring wheat, variety Concord	Canada	commercial (not further specified)	1981	natural	**DON**	570	Bran: ~ +84	Germ: ~ -7	Low-grade flour, millfeed: ~ +207<br>Tail flour, millfeed: ~ +244<br>5th middling flour, millfeed (red dog): ~ +193	–	
Experimental milling in an Allis Chalmers laboratory mill	Batch of hard red spring wheat, variety Concord	Canada	commercial (not further specified)	1981	natural	**DON**	1,190	Bran: ~ +48	–	Shorts: ~ +82<br>Red dog: ~ +105	–	

Industrial milling in Korean mill	Batch composed of 55% of USA-grown soft white winter wheat, 40.5% of USA-grown hard red spring wheat, and 4.5% of Korean wheat	USA/Korea	commercial (not further specified)	1985 or earlier	natural	**DON**	68	Bran: +30	–	Middlings: ~ +28<br>Shorts: ~ +50	–	[12]
						**ZEN**	1	Bran: ~ +100	–	Middlings: ~ +100<br>Shorts: ~ +100	–	
Experimental milling in a Bühler test mill	Wheat *(not further specified)*	Korea	(not specified)	1985	natural	**DON**	310	Bran: ~ +51	–	Red dog: ~ +64<br>Shorts: ~ +87	–	
						**ZEN**	2,047	Bran: ~ +87	–	Red dog: ~ +58<br>Shorts: ~ +134	–	
Experimental milling in an Allis Chalmers laboratory mill combined with an additional purifier (sifter unit)	Canada Eastern White Winter wheat; batch 1: unsorted batch	Canada	commercial (not further specified)	1989	natural	**DON**	7,960	–	–	Millfeed: ~ +10	–	[13]
	", batch 1: third-dense gravity table fraction						5,660	–	–	Millfeed: ~ +25	–	
	", batch 1: second-dense gravity table fraction						1,430	–	–	Millfeed: ~ +60	–	
	", batch 1: densest gravity table fraction						870	–	–	Millfeed: ~ +90	–	
	Canada Eastern White Winter wheat, batch 2: unsorted batch	Canada	commercial (not further specified)	1989	natural		3,730	–	–	Millfeed: ~ +60	–	
	", batch 2: third-dense gravity table fraction						9,340	–	–	Millfeed: ~ +7	–	

	", batch 2: second-dense gravity table fraction						1,810	–	–	Millfeed: ~ +61	–	
	", batch 2: densest gravity table fraction						560	–	–	Millfeed: ~ +109	–	
	Canada Eastern White Winter wheat, batch 3: unsorted batch						1,890	–	–	Millfeed: ~ +61	–	
	", batch 3: third-dense gravity table fraction	Canada	commercial (not further specified)	1989	natural		1,270	–	–	Millfeed: ~ +46	–	
	", batch 3: second-dense gravity table fraction						400	–	–	Millfeed: ~ +93	–	
	", batch 3: densest gravity table fraction						230	–	–	Millfeed: ~ +109	–	
Experimental milling in a laboratory Brabender mill (Quadrumat Junior)	15 varieties of spring wheat	Japan (Hokkaido prefecture)	experimental (not further specified)	2004-2007	natural	**DON**	mean: ca. 6,700	Bran: mean: +80	–	–	Initial level: estimated mean value; increase in bran according to linear regression analysis	[14]
							20	Bran: ~ +64	–	–	Data for the least and for the most contaminated varieties; more data points are available in the study	
							13,412	Bran: ~ +12	–	–		

Experimental milling in a laboratory Bühler mill (MLU-202)	Hard red winter wheat	USA (Nebraska)	(not specified)	1982	natural	**DON**	7,900	Bran: ~ +170	–	Shorts: ~ +122	–	[15]
	Hard red winter wheat	USA (Nebraska)	(not specified)	1982	natural		6,400	Bran: ~ +114	–	Shorts: ~ +42	–	
	Hard red winter wheat	USA (Missouri)	(not specified)	1982	natural		7,800	Bran: ~ +138	–	Shorts: ~ +97	–	
	Hard red winter wheat	USA (Missouri)	(not specified)	1982	natural		7,100	Bran: ~ +59	–	–	–	
	Hard red winter wheat	USA (Kansas)	(not specified)	1982	natural		8,600	Bran: ~ +95	–	Shorts: ~ +6	–	
Experimental milling in a Bühler mill	27 batches of hard red winter wheat	USA (Kansas)	(not specified)	1993	natural	**DON**	mean: 2,800	Bran: mean: ~ +21	–	Shorts: mean: ~ +11	<u>Overall</u>: Individual data were not provided in the study	[16]
						**ZEN**	mean: 950	Bran: mean: ~ +116	–	Shorts: mean: ~ +79		
Experimental milling in a laboratory Bühler mill (MLU-202)	Soft wheat variety Norin 61	Japan (Kyushu)	experimental (not further specified)	2008	inoculation in the field with two *Fusarium graminearum* strains	**DON**	1,890	Bran: ~ +109	–	Shorts: ~ +85	–	[17]
	Soft wheat variety Chikugoizumi	Japan (Kyushu)	experimental (not further specified)	2008	inoculation in the field with two *Fusarium graminearum* strains		900	Bran: ~ -19		Shorts: ~ +74	–	
	Soft wheat variety Norin 61	Japan (Kyushu)	experimental (not further specified)	2008	inoculation in the field with two *Fusarium graminearum* strains	**ZEN**	760	Bran: ~ +36	–	Shorts: ~ +271	–	
	Soft wheat variety Chikugoizumi	Japan (Kyushu)	experimental (not further specified)	2008	inoculation in the field with two *Fusarium graminearum* strains		120	Bran: ~ +67 (n.s.)		Shorts: ~ +142	–	

Experimental milling in a laboratory Bühler mill (MLU-202)	Batch composed of wheat varieties Chikugoizumi and Minaminokaori	Japan (Kyushu)	experimental (not further specified)	2008	inoculation in the field with two *Fusarium graminearum* isolates	**DON**	896	Bran: -19	–	Shorts: +74	–	[18]
Experimental milling in a laboratory Bühler mill (MLU-202)	Different batches of wheat variety Chikugoizumi	Japan (Kyushu)	experimental (not further specified)	2007/ 2008	inoculation in the field with *Fusarium graminearum*	**DON**	900	Bran: ~ -19	–	Shorts: ~ +73	–	[19]
							5,270	Bran: ~ +37	–	Shorts: ~ +80	–	
Experimental milling in a laboratory Bühler mill	Wheat variety Meritto	Czech Republic	conventional	2009	natural	**DON**	182	Bran: ~ +115	–	–	–	[20]
	Wheat variety Akteur	Czech Republic	conventional	2009	natural		198	Bran: ~ +107	–	–	–	
	Wheat variety Eurofit	Czech Republic	conventional	2009	natural		154	Bran: ~ +16	–	–	–	
	Wheat variety Meritto	Czech Republic	conventional (incl. fungizide treatment)	2009	natural		176	Bran: ~ +14	–	–	–	
	Wheat variety Akteur	Czech Republic	conventional (incl. fungizide treatment)	2009	natural		183	Bran: ~ +78	–	–	–	
	Wheat variety Eurofit	Czech Republic	conventional (incl. fungizide treatment)	2009	natural		82	Bran: ~ +12	–	–	–	
	Wheat variety Meritto	Czech Republic	conventional (incl. fungizide treatment)	2009	natural		90	Bran: ~ +119	–	–	–	
	Wheat variety Akteur	Czech Republic	conventional (incl. fungizide treatment)	2009	natural		123	Bran: ~ +105	–	–	–	

	Wheat variety Eurofit	Czech Republic	conventional (incl. fungizide treatment)	2009	natural		125	Bran: ~ +15	–	–	–	
	Wheat variety Meritto	Czech Republic	organic	2009	natural		253	Bran: ~ +24	–	–	–	
	Wheat variety Akteur	Czech Republic	organic	2009	natural		354	Bran: ~ +25	–	–	–	
	Wheat variety Eurofit	Czech Republic	organic	2009	natural		191	Bran: ~ +20	–	–	–	
	Wheat variety Meritto	Czech Republic	experimental (not further specified)	2009	inoculation in the field with *Fusarium culmorum* and *Fusarium graminearum*		1,700	Bran: ~ +28	–	–	–	
	Wheat variety Akteur	Czech Republic	experimental (not further specified)	2009	inoculation in the field with *Fusarium culmorum* and *Fusarium graminearum*		1,586	Bran: ~ +150	–	–	–	
	Wheat variety Eurofit	Czech Republic	experimental (not further specified)	2009	inoculation in the field with *Fusarium culmorum* and *Fusarium graminearum*		1,053	Bran: ~ +47	–	–	–	
Experimental milling in a laboratory Bühler mill (MLU-202)	Canadian Western Red Spring wheat, variety Sinton	Canada (South-eastern Manitoba)	(not specified)	1984	natural	**DON**	~10,900	Bran: +133	–	Shorts: ~ +57	–	[21]
	Canadian Western Amber Durum wheat, variety Coulter	Canada (South-eastern Manitoba)	(not specified)	1984	natural		~8,880	Bran: +50	–	Shorts: ~ +37	–	

Experimental milling in a laboratory Bühler mill (MLU-202)	Wheat variety Sulamit	Czech Republic	conventional	2005	natural	**DON**	456	Bran: ~ +114	–	–	–	[22]
	Wheat variety Sulamit	Czech Republic	organic	2005	natural		59	Bran: ~ +98	–	–	–	
	Wheat variety Ebi	Czech Republic	conventional	2005	natural		38	Bran: ~ +137	–	–	–	
	Wheat variety Ebi	Czech Republic	conventional	2005	inoculation in the field with *Fusarium culmorum* and *Fusarium graminearum*		1,413	Bran: ~ +73	–	–	–	
Experimental milling in a laboratory Bühler mill (MLU-202)	Wheat variety Alex	Romania	(not specified)	2009	post-harvest inoculation with a *Fusarium graminearum* isolate	**DON**	~155	Bran: ~ +122	–	Short: ~ +114	–	[23]
Experimental milling in a laboratory Bühler mill (MLU-202)	Wheat variety Wanmai 50	China (Anhui Province)	(not specified)	2013	natural	**DON**	1,690	Bran: ~ +21	–	Shorts: ~ +74	–	[24]
Experimental milling in a pneumatic laboratory Bühler mill (MLU 220)	Ontario soft white winter wheat	USA (Ontario)	commercial (not further specified)	(not specified)	natural	**DON**	939	Bran: +9	–	Shorts: +25	–	[25]
Experimental milling in a laboratory Bühler mill	Wheat (not further specified)	Japan (Hyogo Prefecture)	(not specified)	1984	natural	**DON**	168	Bran: +172	–	Shorts: +232	–	[26]
						**ZEN**	8	Bran: +138	–	Shorts: +188	–	
Experimental milling in a pilot-scale Brabender mill	Wheat (not further specified)	Brazil (Southern)	experimental (not further specified)	2013	artificial (grain was contaminated with	**DON**	499	Bran (incl. shorts): +26	–	–	–	[27]
							747	Bran (incl. shorts): +17	–	–	–	

(Quadrumat Senior)					screenings of the cleaning and pre-cleaning process of *Fusarium*-susceptible cultivars incl. light and shriveled grains)		1,225	Bran (incl. shorts): +43	–	–	–	
							2,748	Bran (incl. shorts): +24	–	–	–	
							5,985	Bran (incl. shorts): +24	–	–	–	
						**ZEN**	28	Bran (incl. shorts): +127	–	–	–	
							39	Bran (incl. shorts): +58	–	–	–	
Experimental milling in a pilot-scale Brabender mill (Quadrumat Senior)	Wheat variety BRS Parrudo; differently cleaned batch	Brazil (Southern)	commercial (not further specified)	2014	natural	**DON**	4,080	Bran (incl. shorts): +28	–	–	–	[28]
							ca. 1,050	Bran (incl. shorts): +53	–	–	–	
							454	Bran (incl. shorts): +50	–	–	–	
	Wheat variety BRS 374; differently cleaned batch	Brazil (Southern)	commercial (not further specified)	2014	natural		2,038	Bran (incl. shorts): -19	–	–	–	
							ca. 1,300	Bran (incl. shorts): -2	–	–	–	
							515	Bran (incl. shorts): -11	–	–	–	
Experimental milling in a pilot-scale Brabender mill (Quadrumat Senior)	Wheat different varieties (not specified)	Brazil (Southern)	(not specified)	2014	natural	**DON**	ca. 2,400	Bran: ~ +8	–	–	–	[29]
							737	Bran: ~ +76	–	–	–	
							ca. 1,400	Bran: ~ +25	–	–	–	
							ca. 2,400	Bran: ~ +22	–	–	–	
							2,866	Bran: ~ -4	–	–	–	
							ca. 1,800	Bran: ~ +47	–	–	–	
							ca. 1,500	Bran: ~ +17		–	–	

Experimental milling in a pilot-scale Brabender mill (Quadrumat Senior)	30 batches of wheat (not further specified)	Brazil, Argentina, Uruguay	commercial (not further specified)	2012, 2013	natural	**DON**	mean: 1,098 (range: ca. 300–3,500)	Bran: mean: +73	–	Shorts: mean: +35	–	[30]
Experimental milling in a laboratory Bühler mill (MLU-202)	Winter wheat variety Eurofit	(not specified)	conventional	(not specified)	natural	**DON**	246	Bran: ~ +42	–	Shorts: ~ +69	–	[31]
	Winter wheat variety Eurofit	(not specified)	organic	(not specified)	natural		318	Bran: ~ +74	–	Shorts: ~ +81	–	
Experimental milling using a laboratory Bühler mill (MLU-202) + Namad purifier for separation of semolina	Different batches of durum wheat, variety Levante	Italy	experimental (conventional)	(not specified)	inoculation in the field with a *Fusarium sporotrichioides* isolate	**T-2**	13	Bran: ~ +369	–	Fine middlings: ~ +8<br>Red dog: ~ +392	–	[32]
							43	Bran: ~ +247	–	Fine middlings: ~ -12<br>Red dog: ~ +298	–	
							35	Bran: ~ +751	–	Fine middlings: ~ +23<br>Red dog: ~ +474	–	
							136	Bran: ~ +157	–	Fine middlings: ~ -63<br>Red dog: ~ +138	–	
							71	Bran: ~ +279	–	Fine middlings: ~ -32<br>Red dog: ~ +155	–	
							184	Bran: ~ +707	–	Fine middlings: ~ -15<br>Red dog: ~ +165	–	
						**HT-2**	55	Bran: ~ +365	–	Fine middlings: ~ -42<br>Red dog: ~ +229	–	
							110	Bran: ~ +591	–	Fine middlings: ~ -22<br>Red dog: ~ +384	–	
							169	Bran: ~ +535	–	Fine middlings: ~ -40<br>Red dog: ~ +238	–	
							374	Bran: ~ +437	–	Fine middlings: ~ -71<br>Red dog: ~ +138	–	
							943	Bran: ~ +392	–	Fine middlings: ~ -60<br>Red dog: ~ +190	–	
							2,221	Bran: ~ +1,189	–	Fine middlings: ~-48<br>Red dog: ~ +250	–	

Industrial milling in a UK mills (might contain some cleaning)	Different batches of wheat (not further specified)	(not specified)	commercial (not further specified)	2004-2007	natural	**T-2**	<10	Bran: ≳ +180	Germ: ca. 0	–	–	[33]
							<10	Bran: ≳ +250	Germ: ≳ +30	–	–	
							<10	Bran: ≳ +260	Germ: ≳ +10	–	–	
							<10	Bran: ≳ +140	–	–	–	
							<10	Bran: ≳ +120	Germ: ca. 0	–	–	
							<10	Bran: ≳ +190	Germ: ≳ +110	–	–	
							12	Bran: ~ +542	Germ: ~ +92	–	–	
							<10	Bran: ≳ +330	Germ: ≳ +10	–	–	
							10	Bran: ~ +470	Germ: ~ +30	–	–	
							<10	Bran: ≳ +460	Germ: ≳ +240	–	–	
							<10	Bran: ≳ +390	Germ: ca. 0	–	–	
							<10	Bran: ≳ +80	Germ: ≳ +20	–	–	
							<10	Bran: ≳ +310	Germ: ≳ +210	–	–	
							<10	Bran: ≳ +120	Germ: ≳ +60	–	–	
							<10	Bran: ≳ +260	Germ: ca. 0	–	–	
							<10	Bran: ≳ +520	Germ: ≳ +240	–	–	
						**HT-2**	<10	Bran: ≳ +420	Germ: ca. 0	–	–	
							11	Bran: ~ +764	Germ: ~ +164	–	–	
							12	Bran: ~ +450	Germ: ~ +42	–	–	
							<10	Bran: ≳ +260	–	–	–	
							<10	Bran: ≳ +150	Germ: ca. 0	–	–	
							<10	Bran: ≳ +140	Germ: ≳ +40	–	–	
							<10	Bran: ≳ +560	Germ: ≳ +40	–	–	
							<10	Bran: ≳ +310	Germ: ca. 0	–	–	
							<10	Bran: ≳ +340	Germ: ca. 0	–	–	
							<10	Bran: ≳ +410	Germ: ≳ +160	–	–	
							<10	Bran: ≳ +290	Germ: ca. 0	–	–	
							<10	Bran: ≳ +30	Germ: ca. 0	–	–	
							<10	Bran: ≳ +260	Germ: ≳ +130	–	–	
							<10	Bran: ≳ +110	Germ: ≳ +30	–	–	

							<10	Bran: $\gtrsim$ +360	Germ: ca. 0	–	–	
							<10	Bran: $\gtrsim$ +560	Germ: $\gtrsim$ +90	–	–	
Experimental milling in a pilot-scale Bühler mill, incl. cleaning by a Carter-Day Dockage Tester	Different batches of wheat variety Hereward (baking quality wheat)	(not specified)	commercial (not further specified)	(not specified)	post-harvest inoculation with *Penicillium verrucosum*	**OTA**	6.1	Bran: ~ +287	–	Bran flour: ~ -3<br>Shorts (offal): ~ +987<br>Shorts (offal) flour: ~ +51	–	
							42	Bran: ~ +186	–	Bran flour: ~ +14<br>Shorts (offal): ~ +384<br>Shorts (offal) flour: ~ +150	–	
Experimental milling in a pilot-scale Bühler mill, incl. cleaning by a Carter-Day Dockage Tester and abrassive scouring by a Westrup LA-H scourer (lower surface removal)	Different batches of wheat variety Hereward (baking quality wheat)	(not specified)	commercial (not further specified)	(not specified)	post-harvest inoculation with *Penicillium verrucosum*	**OTA**	6.1	Bran: ~ +48	–	Bran flour: ~ +15<br>Shorts (offal): ~ +523<br>Shorts (offal) flour: ~ +30	–	
							42	Bran: ~ +179	–	Bran flour: ~ -30<br>Shorts (offal): ~ +194<br>Shorts (offal) flour: ~ +269	–	[34]
Experimental milling in a pilot-scale Bühler mill, incl. cleaning by a Carter-Day Dockage Tester and abrassive scouring by a Westrup LA-H scourer (higher surface removal)	Different batches of wheat variety Hereward (baking quality wheat)	(not specified)	commercial (not further specified)	(not specified)	post-harvest inoculation with *Penicillium verrucosum*	**OTA**	6.1	Bran: ~ +84	–	Bran flour: ~ -39<br>Shorts (offal): ~ +438<br>Shorts (offal) flour: ~ -31	–	
							42	Bran: ~ +129	–	Bran flour: ~ -36<br>Shorts (offal): ~ +359<br>Shorts (offal) flour: ~ +164	–	

Experimental milling in a laboratory mill (LRMM8040-3-D)	Different batches of wheat variety Shijiazhuang 8	China (Hebei Province)	(not specified)	(not specified)	post-harvest inoculation with *Aspergillus ochraceus* isolates	**OTA**	93	+66	–	Shorts: ~ +112	–	[35]
							249	+43	–	Shorts: ~ +116	–	

[a] If not mentioned otherwise, the milling procedures do not include cleaning of whole grain.

[b] Change in the mycotoxin concentration compared to the concentration in the unprocessed grain. Negative values: reduction; positive values: increase; '$\gtrsim$' represents an approximately equal or more pronounced change.

DON: deoxynivalenol; OTA: ochratoxin A; ZEN: zearalenone.

incl.: including; LOD: limit of detection; n.d.: mycotoxin not detected in the product; n.s.: not statistically significant according to original study, change is presented in Figure as '0'.

~: Approximate values that were calculated for this overview by using the data provided in the cited literature.

Table S2. Effect of maize dry milling on mycotoxin concentrations in by-products.

Milling procedure/ technology	Type of maize and variety	Origin	Type of cultivation	Harvest year	Type of contamination	Myco-toxin	Initial level in whole kernels (µg/kg)	Change [%] [b] – bran	Change [%] [b] – germ	Change [%] [b] – other by-products (animal feed flour, hominy feed etc.)	Comments	Refer-ence
Dehulling by a BECO hand mill	White shelled dent maize, variety MM 604	Zambia	(not specified)	(not specified)	post-harvest inoculation with *Aspergillus*	**AFB1**	911	Bran: ~ +34	–	–	–	[36]
						**AFB2**	96	Bran: ~ +25 (n.s.)	–	–	–	
						**AFG1**	929	Bran: ~ +20 (n.s.)	–	–	–	
						**AFG2**	155	Bran: ~ -7 (n.s.)	–	–	–	
Dehulling by a traditional African mortar-and-pestle technique (incl. addition of a wetting agent) followed by drying, sieving, and manual winnowing	Different batches of maize (not further specified)	Kenya	(not specified)	(not specified)	natural	**AFB1**	20	Bran + fines (≤ 2 mm): ca. +600	–	–	Circa values are taken from diagram; more data points are available in the study	[37]
							25	Bran + fines: ca. +400	–	–		
							50	Bran + fines: ca. +130	–	–		
							50	Bran + fines: ca. +470	–	–		
							65	Bran + fines: ca. +300	–	–		
							100	Bran + fines: ca. +200	–	–		
							150	Bran + fines: ca. +200	–	–		
							200	Bran + fines: ca. 0	–	–		
							220	Bran + fines: ca. +170	–	–		
							270	Bran + fines: ca. +130	–	–		
Dehulling in a hammer mill/dehuller (at elevated moisture) followed by winnowing to simulate traditional African processes	Open pollinated maize variety OPV623	Malawi	(not specified)	(not specified)	post-harvest inoculation with aflatoxinogenic *Aspergillus* species	**AFB1**	mean: 48 (range: 8.9–87)	Bran: mean: +88	–	–	Linear regression with $R^2 = 0.996$ (dilution series using a contaminated batch and uncontaminated grain)	[38]
Industrial dry milling (might contain cleaning)	92 batches of maize (not further specified)	Argentina	commercial (not further specified)	2002-2004	natural	**Total AFs**	mean: 2.6 (range: n.d.–4.8)	–	–	Animal feed flour (mainly bran + germ): mean: +188	<u>Overall</u>: Individual data were not	[39]

						**Total FBs**	mean: 2,610 (range: 337–10,613)	–	–	Animal feed flour: mean: +217	provided in the study	
Industrial dry milling coupled with wet degermination of kernels (at 22% moisture) by a conical de-germinator	1–2 batches of FAO 600 class maize hybrids with flinty endosperm	(not specified)	commercial (not further specified)	(not specified)	natural	**AFB1**	39.5	Bran: +379	Germ: +629	Animal feed flour (bran, undersized flour + milled cleaning waste): ~ +456	The changes in the mycotoxin levels of the animal feed flour (although it covers cleaning waste) is also related to cleaned maize.	[40]
							3.3	Bran: +127	Germ: +197	Animal feed flour: ~ +3		
						**AFB2**	1.8	Bran: ~ +306	Germ: ~ +828	Animal feed flour: ~ +389		
						**AFG1**	14	Bran: ~ +207	Germ: ~ +489	Animal feed flour: ~ +249		
							1.1	Bran: ~ +255	Germ: ~ +373	Animal feed flour: ~ 0		
						**AFG2**	0.7	Bran: ~ +57	Germ: ~ +614	Animal feed flour: ~ +143		
						**FB1**	5,862	Bran: +167	Germ: ~ +99	Animal feed flour: ~ +390		
							4,770	Bran: +50	Germ: ~ -30	Animal feed flour: ~ +236		
						**FB2**	2,975	Bran: ~ +88	Germ: ~ +46	Animal feed flour: ~ +392		
							2,360	Bran: ~ +8	Germ: ~ -48	Animal feed flour: ~ +246		
Dry milling (probably commercial milling incl. cleaning)	Maize (not further specified)	(not specified)	(not specified)	(not specified)	probably natural	**AF(s)**	51	–	Germ: +110	Hominy feed: +140	–	[41]
						**ZEN**	1,000	–	Germ: +300	Hominy feed: +200	–	

Industrial dry milling coupled with cleaning (dry de-stoner, scourer, aspirator) and wet degermination of kernels (at 20% moisture)	Maize (not further specified)	(not specified)	coventional	(not specified)	natural	**AFB1**	0.93	Bran: +444	Germ: +239	Animal feed flour: +253	–	[42]
	Maize (not further specified)	(not specified)	organic	(not specified)	natural		2.8	Bran: +811	Germ: +274	Animal feed flour: +650	–	
	Maize (not further specified)	(not specified)	coventional	(not specified)	natural	**AFB2**	0.15	Bran: ~ +347	Germ: ~ +87	Animal feed flour: ~ +133	–	
	Maize (not further specified)	(not specified)	organic	(not specified)	natural		<0.15	Bran: > ~ +1207	Germ: > ~ +347	Animal feed flour: > ~ +480	–	
	Maize (not further specified)	(not specified)	organic	(not specified)	natural	**AFG1**	0.38	Bran: ~ +387	Germ: ~ -5	Animal feed flour: ~ +139	–	
	Maize (not further specified)	(not specified)	coventional	(not specified)	natural	**ZEN**	89.6	Bran: +203	Germ: +139	Animal feed flour: +251	–	
	Maize (not further specified)	(not specified)	organic	(not specified)	natural		9.8	Bran: +303	Germ: +214	Animal feed flour: +324	–	
Industrial dry milling coupled with cleaning (dry de-stoner, scourer, aspirator) and wet degermination of kernels (at 20% moisture)	2 lots of one batch of maize (not further specified)	(not specified)	commercial (not further specified)	(not specified)	natural	**FB1**	4,540	Bran: ~ +56	Germ: ~ +96	Animal feed flour (undersized flour + milled broken kernels, cob, etc.): ~ +106	–	[43]
							5,090	Bran: ~ +59	Germ: ~ +88	Animal feed flour: ~ +35	–	

Industrial dry milling in an Italian mill (involving rollermills and plansifters) coupled with dry degermination (by breakers, plansifters, and gravity tables)	Maize (not further specified)	(not specified)	commercial (not further specified)	(not specified)	natural	**Total FBs**	926	–	Germ: ~ +1 (n.s.)	Middlings (animal feed flour): ~ +566	Here, the middlings contain 'usuable waste' from the cleaning process that was milled to animal feed. The change in the fumonisin level of this by-product is also related to cleaned maize. (Cleaning did not cause a significant reduction in the fumonisin level of the cleaned grain.)	[44]
Commercial dry milling in an Italian mill (involving rollermills, sifters, and aspirator) coupled with cleaning (dry de-stoner, scourer, aspirator) and dry degermination (by breaker, plansifter, and gravity table)	24 batches of maize (not further specified)	(not specified)	commercial (not further specified)	likely 2002-2006	natural	**FB1**	mean: 4,580 (range: 273–8,480)	–	Germ: mean: ~ -25 (n.s.)	Animal meal (bran + undersized flour): mean: ~ +149	Individual data were not provided in the study	[45]

Industrial dry milling in an Italian mill (involving rollermills, plansifters, flour purifiers) coupled with dry degermination (by impact degermer, plansifters, and gravity separators)	9 batches of 7 different maize varieties and mixtures thereof	Italy (North-western)	commercial (not further specified)	2011-2013	natural	**FB1+FB2**	mean: 725	–	Germ: mean: ~ -27	Animal feed flour (bran + undersized flour): mean: ~ +257	Overall: Individual data were not provided in the study	[46]
						**FB1**	mean: 534	–	Germ: mean: ~ -26	Animal feed flour: mean: ~ +222		
						**FB2**	mean: 191	–	Germ: mean: ~ -31	Animal feed flour: mean: ~ +357		
Industrial dry milling in an Italian mill using a Beall type degermer (wet degermination) combined with plansifters and gravity separators	9 batches of 7 different maize varieties and mixtures thereof	Italy (North-western)	commercial (not further specified)	2011-2013	natural	**FB1+FB2**	mean: 725	–	Germ: mean: ~ -30	Animal feed flour: mean: ~ +326		
						**FB1**	mean: 534	–	Germ: mean: ~ -28	Animal feed flour: mean: ~ +287		
						**FB2**	mean: 191	–	Germ: mean: ~ -35	Animal feed flour: mean: ~ +433		
Industrial dry milling with dry degermination and refining	4 batches of maize, varieties: Pioneer P1543, Pioneer P1547, Pioneer PR32B10, Pioneer P0722	Italy (North-western)	commercial (not further specified)	2011, 2012, 2014	natural	**FB1+FB2**	mean: 818	–	Germ: ~ -12	Animal feed flour: ~ +358	Overall: Individual data were not provided in the study	[47]
Industrial dry milling with wet degermination using a Beall type degermer	4 batches of maize, varieties: Pioneer P1543, Pioneer P1547, Pioneer PR32B10, Pioneer P0722	Italy (North-western)	commercial (not further specified)	2011, 2012, 2014	natural	**FB1+FB2**	mean: 818	–	Germ: ~ -4	Animal feed flour: ~ +538		

Commercial dry milling (might include some cleaning)	Different batches of maize (not further specified)	(not specified)	commercial (not further specified)	1992 or earlier	natural	**Total FBs**	600	Bran: ~ +200	Germ: ~ -83	–	–	
							3,300	Bran: ~ -33	Germ: ~ -64	–	–	
							300	Bran: ~ +433	Germ: ~ +233	–	–	
							<100	Bran: ~ +1,900	Germ: ~ +800	–	–	
							3,200	Bran: ~ -9	Germ: ~ -84	–	–	
							200	Bran: ~ +1,500	Germ: ~ +200	–	–	
							3,500	Bran: ~ -40	Germ: ~ -74	–	–	
							100	Bran: ~ +2,000	Germ: ~ +1,900	–	–	
							300	Bran: ~ +467	Germ: ~ 0	–	–	
							100	Bran: ~ +1,700	Germ: ~ +600	–	–	
							3,300	Bran: ~ -39	Germ: ~ -82	–	–	
							700	Bran: ~ +114	Germ: ~ -29	–	–	
Experimental dry milling using a laboratory horizontal drum degermer (wet degermination at 20% moisture) combined with sieving, aspiration, and manual separation of germs	White food-grade dent maize	USA (Nebraska)	commercial (not further specified)	1994	natural	**FB1**	300	Bran: ~ +400	Germ: ~ +270	Fines: ~ +385	All changes: very approximate data based on data taken from diagrams	[48]
	Blue food-grade dent maize	(not specified)	commercial (not further specified)	1995	natural		3,900	Bran: ~ +110	Germ: ~ -20 (n.s.)	Fines: ~ +390		
	Yellow food-grade dent maize	(not specified)	commercial (not further specified)	1995	natural		25,400	Bran: ~ +160	Germ: ~ -40 (n.s.)	Fines: ~ +275		
	White food-grade dent maize	USA (Nebraska)	commercial (not further specified)	1994	natural	**FB2**	270	Bran: ~ +190	Germ: ~ +300	Fines: ~ +165		
	Blue food-grade dent maize	(not specified)	commercial (not further specified)	1995	natural		1,600	Bran: ~ +50	Germ: ~ +25 (n.s.)	Fines: ~ +325		
	Yellow food-grade dent maize	(not specified)	commercial (not further specified)	1995	natural		8,900	Bran: ~ +125	Germ: ~ -40 (n.s.)	Fines: ~ +315		

Industrial dry milling in an Argentinean mill	13–14 batches of maize (not further specified)	(not specified)	commercial (not further specified)	1998-1999	natural	**FB1**	mean: 1,540 (range: 54–5,960)	–	–	Bran + germ: mean: ~ +173	<u>Overall</u>: Individual data were not provided in the study	[49]
						**FB2**	mean: 716 (range: 17–3,080)	–	–	Bran + germ: mean: ~ +181		
						**FB3**	mean: 152 (range: 8–669)	–	–	Bran + germ: mean: ~ +194		
Industrial dry milling in an Brazilian mill	Different batches (with 20 subsamples each) of a non-transgenic maize (not further specified)	Brazil	commercial (not further specified)	2014	natural	**FB1+FB2**	mean: 579 (range: n.d.–1,822)	Bran: mean: +88	Germ: mean: +222	–	<u>Overall</u>: Individual subsample data were not provided in the study	[50]
				2015	natural		mean: 1,309 (range: 304–2,540)	Germ: mean: +163	Germ: mean: +211	–		
				2014	natural	**FB1**	mean: 481 (range: n.d.–1,441)	Bran: mean: ~ +95	Germ: mean: ~ +166	–		
				2015	natural		mean: 957 (range: 304–1,865)	Germ: mean: ~ +442	Germ: mean: ~ +500	–		
				2014	natural	**FB2**	mean: 270 (range: n.d.–380)	Bran: mean: ~ +24	Germ: mean: ~ +94	–		

				2015	natural		mean: 440 (range: n.d.–704)	Germ: mean: ~ +244	Germ: mean: ~ +337	–		
Industrial dry milling in an Brazilian mill	2 batches (with 20 subsamples each) of a hybrid from Pioneer DuPont and P30F53**YHR** (transgenic maize), semi-flint maize	Brazil (Paraná State)	commercial (not further specified)	2015+ 2016	natural	**FB1+FB2**	mean: 1,130 (range: 155–2,860)	Bran: mean: +115	Germ: mean: +120	–	<u>Overall</u>: Individual data were not provided in the study	[51]
	2 batches (with 20 subsamples each) of a hybrid from Pioneer DuPont and P30F53 (non-transgenic maize), semi-flint maize	Brazil (Paraná State)	commercial (not further specified)	2015+ 2016	natural		mean: 920 (range: 120–2,540)	Bran: mean: +126	Germ: mean: +217	–		
Industrial dry milling in 2–3 South African mills	Maize (not further specified) [mill A]	(not specified)	commercial (not further specified)	(not specified)	natural	**FB1+FB2**	mean: 567 (range: 69–1,171)	–	–	Hominy feed: mean: +179	<u>Overall</u>: Individual data were not provided in the study	[52]
	Maize (not further specified) [mill B]	(not specified)	commercial (not further specified)	(not specified)	natural		mean: 302.3 (range: 0–90)	–	–	Hominy feed: mean: +103		

	Maize (not further specified) [mill A]	(not specified)	commercial (not further specified)	(not specified)	natural	**DON**	mean: 112 (range: 0–201)	–	–	Hominy feed: mean: +465		
	Maize (not further specified) [mill B]	(not specified)	commercial (not further specified)	(not specified)	natural		mean: 35 (range: 5–88)	–	–	Hominy feed: mean: +779		
	Maize (not further specified) [mill C]	(not specified)	commercial (not further specified)	(not specified)	natural		mean: 90 (range: 43–183)	–	–	Hominy feed: mean: +484		
	Maize (not further specified) [mill A]	(not specified)	commercial (not further specified)	(not specified)	natural	**ZEN**	mean: 3.7 (range: 0–11)	–	–	Hominy feed: mean: +1,324		
	Maize (not further specified) [mill B]	(not specified)	commercial (not further specified)	(not specified)	natural		mean: 6.3 (range: 0–19)	–	–	Hominy feed: mean: +249		
Experimental dry milling in a modified Bühler mill (MLU-202) coupled with dry degermination of kernels using a Grainman rice polisher	5 composite batches containing maize of different qualities (not further specified)	South Africa	(not specified)	2010	natural	**FB1+FB2**	mean: 413 (range: 105–1,268)	–	–	Hominy feed: mean: +180		
						**DON**	mean: 329 (range: 68–787)	–	–	Hominy feed: mean: +140		
						**ZEN**	mean: 93 (range: 8–307)	–	–	Hominy feed: mean: +163		
Experimental milling in a laboratory Bühler mill (MLU-202)	Maize (not further specified)	Italy	(not specified)	2012	natural	**Total FBs**	7,125	Bran: +69	–	–	–	[53]

Industrial dry milling in a Romanian mill (involving roller mills, plansifter, purifier) coupled with degermination (degermer, plansifter, gravity tables)	Maize (not further specified)	Romania (South eastern)	commercial (not further specified)	(not specified)	natural	**Total FBs**	1334	–	–	Hominy feed: ~ +123	–	[54]
						**T-2**	760	–	–	Hominy feed: ~ +150	–	
Industrial dry milling	1–2 batches of maize (not further specified)	(not specified)	commercial (not further specified)	(not specified)	natural	**DON**	472	Bran: ~ +1,316	Germ: ~ +439	–	–	[55]
							270	Bran: ~ +595	Germ: ~ +228	–	–	
						**ZEN**	123	Bran: ~ +332	Germ: ~ +505	–	–	
							181	Bran: ~ +372	Germ: ~ +97	–	–	
						**T-2**	6	Bran: ~ +183	Germ: ~ +567	–	–	
						**HT-2**	5	Bran: ~ +1,180	Germ: ~ +1,540	–	–	
							5	Bran: ~ +1,880	Germ: ~ +280	–	–	
Commercial dry milling (might include cleaning)	Different batches of maize (not further specified)	(not specified)	commercial (not further specified)	(not specified)	natural	**DON**	80	–	–	Germ meal (animal feed; likely incl. bran): ~ +88	–	[56]
							450	–	–	Germ meal: ~ +35	–	
Industrial dry milling in UK mills (might include cleaning)	Different batches of French Dent or Argentine Flint maize	France or Argentina	(not specified)	2004-2007	natural	**T-2**	<10	Bran: $\gtrsim$ +180	Germ: $\gtrsim$ 0	–	–	[33]
							<10	Bran: $\gtrsim$ +250	Germ: $\gtrsim$ +30	–	–	
							<10	Bran: $\gtrsim$ +260	Germ: $\gtrsim$ +10	–	–	
							<10	Bran: $\gtrsim$ +140	Germ: –	–	–	
							<10	Bran: $\gtrsim$ +120	Germ: $\gtrsim$ 0	–	–	
							<10	Bran: $\gtrsim$ +190	Germ: $\gtrsim$ +110	–	–	
							12	Bran: ~ +542	Germ: ~ +92	–	–	
							<10	Bran: $\gtrsim$ +330	Germ: $\gtrsim$ +10	–	–	
							10	Bran: ~ +470	Germ: ~ +30	–	–	
							<10	Bran: $\gtrsim$ +460	Germ: $\gtrsim$ +240	–	–	
							<10	Bran: $\gtrsim$ +390	Germ: $\gtrsim$ 0	–	–	

	<10	Bran: ≳ +80	Germ: ≳ +20	–	–
	<10	Bran: ≳ +310	Germ: ≳ +210	–	–
	<10	Bran: ≳ +120	Germ: ≳ +60	–	–
	<10	Bran: ≳ +260	Germ: ≳ 0	–	–
	<10	Bran: ≳ +520	Germ: ≳ +240	–	–
**HT-2**	<10	Bran: ≳ +420	Germ: ≳ 0	–	–
	11	Bran: ~ +764	Germ: ~ +164	–	–
	12	Bran: ~ +450	Germ: ~ +42	–	–
	<10	Bran: ≳ +260	Germ: –	–	–
	<10	Bran: ≳ +150	Germ: ≳ 0	–	–
	<10	Bran: ≳ +140	Germ: ≳ +40	–	–
	<10	Bran: ≳ +560	Germ: ≳ +40	–	–
	<10	Bran: ≳ +310	Germ: ≳ 0	–	–
	<10	Bran: ≳ +340	Germ: ≳ 0	–	–
	<10	Bran: ≳ +410	Germ: ≳ +160	–	–
	<10	Bran: ≳ +290	Germ: ≳ 0	–	–
	<10	Bran: ≳ +30	Germ: ≳ 0	–	–
	<10	Bran: ≳ +260	Germ: ≳ +130	–	–
	<10	Bran: ≳ +110	Germ: ≳ +30	–	–
	<10	Bran: ≳ +360	Germ: ≳ 0	–	–
	<10	Bran: ≳ +560	Germ: ≳ +90	–	–

[a] If not mentioned otherwise, the milling procedures do not include cleaning of whole grain.

[b] Change in the mycotoxin concentration compared to the concentration in the unprocessed grain. Negative values: reduction; positive values: increase; ">" represents a more pronounced change (in case of negative numbers the absolute value is less than the indicated one).

AF(s): aflatoxin(s) not further specified in the cited study; AFB1/2: aflatoxin B1/2; AFG1/2: aflatoxin G1/2; DON: deoxynivalenol; FB1/2/3: fumonisin B1/2/3; OTA: ochratoxin A; total AFs: aflatoxins B1+B2+G1+G2; total FBs: fumonisins B1+B2+B3; ZEN: zearalenone.

incl.: including; n.s.: not statistically significant according to original study, change is presented in Figure as '0'.

~: Approximate values that were calculated for this overview by using the data provided in the cited literature.

Table S3. Effect of maize wet milling on mycotoxin concentrations in by-products.

Milling prrocedure/ technology	Type of maize and variety	Origin	Type of cultivation	Harvest year	Type of contamination	Myco-toxin	Initial level in whole kernels (µg/kg)	Change (%) [a] — gluten	Change (%) [a] — fibre	Change (%) [a] — germ	Change (%) [a] — other by-products	Comment(s)	Refe-rence
Laboratory wet milling (incl. steeping of kernels in water for 48 h)	Maize (not further specified)	likely Egypt	commercial (not further specified)	(not specified)	post-harvest inoculation with an *Aspergillus flavus* isolate	**Total AFs**	487	Gluten: ~ +165	–	–	Germ + fiber: ~ -61	In this study, data appear to be aflatoxin amounts (although declared by the author as aflatoxin concentrations). The changes in concentrations presented here are approximate values that were calculated by taking the weight-% of the grain fractions into account.	[57]
						**AFB1**	185	Gluten: ~ +147	–	–	–		
						**AFB2**	111	Gluten: ~ +223	–	–	–		
						**AFG1**	115	Gluten: ~ +57	–	–	–		
						**AFG2**	76	Gluten: ~ +283	–	–	–		

Laboratory wet milling (incl. steeping of kernels in water for 48 h)	Maize (not further specified)	likely Egypt	commercial (not further specified)	(not specified)	post-harvest inoculation with *Aspergillus parasiticus* isolates	**AFB1**	~221	Gluten: ~ +115	–	–	Germ + fiber: ~ -76	In this study, data appears to be AFB1 amounts (although declared by the author as AFB1 concentrations). The changes in concentrations presented here are very approximate values that were calculated by taking the weight-% of the grain fractions published in an earlier study [57] into account.	[58]
Laboratory wet milling (incl. steeping of kernels in dilute solutions of lactic acid and SO_2, pH 4, at 49°C for 48 h)	Maize (not further specified)	(not specified)	(not specified)	(not specified)	post-harvest inoculation with an *Aspergillus flavus* isolate	**AFB1**	638	Gluten: ~ +20	Fiber: ~ +151	Germ: ~ +25	Soluble dry substances from steep and wash water: ~ +284	–	[59]
	Maize (not further specified)	(not specified)	commercial (not further specified)	(not specified)	natural		120	Gluten: ~ +17	Fiber: ~ +183	Germ: ~ +17	Solubles: ~ +408	–	
Commercial wet-milling in three Korean mills	6 batches of maize (not further specified)	USA	commercial (not further specified)	(not specified)	natural	**AFB1**	mean: 0.1 (range: <0.1–0.4)	Gluten: mean: ~ +100 [b]	Fiber: mean: ~ +100 [b]	Germ: mean: ~ +100 [b]	Gluten feed: mean: ~ +100 [b]	Overall: Very approximate mean changes (individual data not provided)	[60]

						**FB1**	mean: 61 (range: >2.4–178)	Gluten: mean: ~ +2,200 [b]	Fiber: mean: ~ +1,235 [b]	Germ: mean: ~ +1,600 [b]	Gluten feed: mean: ~ +4,000 [b]		
						**FB2**	mean: 16 (range: >2.3–53.9)	Gluten: mean: ~ +5,120 [b]	Fiber: mean: ~ +1,655 [b]	Germ: mean: ~ +3,140 [b]	Gluten feed: mean: ~ +2,075 [b]		
						**DON**	mean: 214 (range: <4.1–834)	Gluten: mean: ~ +135 [b]	Fiber: mean: ~ +150 [b]	Germ: mean: ~ +65 [b]	Gluten feed: mean: ~ +555 [b]		
						**ZEN**	mean: 40 (range: <0.2–200)	Gluten: mean: ~ +730 [b]	Fiber: mean: ~ +260 [b]	Germ: mean: ~ +650 [b]	Gluten feed: mean: ~ +195 [b]		
						**T-2**	mean: 2.0 (range: <0.1–10)	Gluten: mean: ~ +510 [b]	Fiber: mean: ~ +60 [b]	Germ: mean: ~ +245 [b]	Gluten feed: mean: ~ +315 [b]		
						**HT-2**	<5.6	Gluten: mean: ~ +175 [b]			Gluten feed: mean: ~ +320 [b]		
Wet milling (likely commercial)	Maize (not further specified)	(not specified)	(not specified)	(not specified)	probably natural	**AFs**	120	Gluten: +20 [b]	Fiber: +180 [b]	Germ: +20 [b]	Steep-water solubles: +410 [b]	–	[41]
						**ZEN**	900	Gluten: +660 [b]	Fiber: +200 [b]	Germ: +90 [b]	Steep-water solubles: +330 [b]	–	
Traditional West-African wet milling (incl. steeping/ fermentation of	Yellow maize	likely Nigeria	commercial (not further specified)	(not specified)	natural	**Total AFs**	588	–	Fiber:<br>48-h steeping: -75 [b]<br>72-h steeping: -71 [b]<br>96-h steeping: -59 [b]	–	–	–	[61]

kernels in water for 48, 72 or 96 h)	Yellow maize	likely Nigeria	commercial (not further specified)	(not specified)	natural	**AFB1**	513	–	Fiber:<br>48-h steeping: -73[b]<br>72-h steeping: -70[b]<br>96-h steeping: -56[b]	–	–	–
	Yellow maize	likely Nigeria	commercial (not further specified)	(not specified)	natural	**AFB2**	75	–	Fiber:<br>48-h steeping: -88[b]<br>72-h steeping: -83[b]<br>96-h steeping: -77[b]	–	–	–
	White maize	likely Nigeria	commercial (not further specified)	(not specified)	natural	**Total FBs**	780	–	Fiber:<br>48-h steeping: -93[b]<br>72-h steeping: -88[b]<br>96-h steeping: -88[b]	–	–	–
	Yellow maize	likely Nigeria	commercial (not further specified)	(not specified)	natural		2,294	–	Fiber:<br>48-h steeping: -95[b]<br>72-h steeping: -94[b]<br>96-h steeping: -94[b]	–	–	
	White maize	likely Nigeria	commercial (not further specified)	(not specified)	natural	**FB1**	483	–	Fiber:<br>48-h steeping: -85[b]<br>72-h steeping: -80[b]<br>96-h steeping: -65[b]	–	–	–
	Yellow maize	likely Nigeria	commercial (not further specified)	(not specified)	natural		1,586	–	Fiber:<br>48-h steeping: -97[b]<br>72-h steeping: -96[b]<br>96-h steeping: -95[b]	–	–	
	White maize	likely Nigeria	commercial (not further specified)	(not specified)	natural	**FB2**	229	–	Fiber:<br>48-h steeping: -93[b]<br>72-h steeping: -87[b]<br>96-h steeping: -83[b]	–	–	–
	Yellow maize	likely Nigeria	commercial (not further specified)	(not specified)	natural		456	–	Fiber:<br>48-h steeping: -91[b]<br>72-h steeping: -87[b]<br>96-h steeping: -89[b]	–	–	

	White maize	likely Nigeria	commercial (not further specified)	(not specified)	natural	**FB3**	68	–	Fiber:<br>48-h steeping: -90 [b]<br>72-h steeping: -86 [b]<br>96-h steeping: -97 [b]	–	–	–	
	Yellow maize	likely Nigeria	commercial (not further specified)	(not specified)	natural		252	–	Fiber:<br>48-h steeping: -96 [b]<br>72-h steeping: -94 [b]<br>96-h steeping: -97 [b]	–	–		
	White maize	likely Nigeria	commercial (not further specified)	(not specified)	natural	**ZEN**	3.3	–	Fiber:<br>48-h steeping: -100 [b]<br>72-h steeping: -86 [b]<br>96-h steeping: -39 [b]	–	–		
	Yellow maize	likely Nigeria	commercial (not further specified)	(not specified)	natural		205	–	Fiber:<br>48-h steeping: -99.6 [b]<br>72-h steeping: -99.6 [b]<br>96-h steeping: -97 [b]	–	–	–	
Wet milling (incl. steeping of kernels in hot water with addition of 0.2% SO_2 for 40–50 h); incl. <u>cleaning</u>	Maize (not further specified)	(not specified)	(not specified)	(not specified)	probably natural	**FB1**	2,590	Gluten: ~ -72 [b]	Fiber: ~ -83 [b]	Germ: ~ -94 [b]	–	–	[62]
						**FB2**	450	Gluten: ~ -38 [b]	Fiber: ~ -44 [b]	Germ: ~ -89 [b]	–	–	
Laboratory wet milling (incl. steeping of kernels in a 0.55% lactic acid, 0.2% sulfurous acid solution at 52°C for 36 h)	Yellow maize	(not specified)	(not specified)	1989	natural	**FB1**	13,900	Gluten: ~ -61 [b]	Fiber: ~ -70 [b]	Germ: ~ -84 [b]	–	Changes are of mean values of two replicate runs	[63]
Commercial (but small-scale) traditional West-African wet milling (incl. steeping/ fermentation of kernels in water for 72 h); might include cleaning	Maize (not further specified)	likely Nigeria	commercial (not further specified)	(not specified)	natural	**FB1**	3,239	–	Fiber: ~ -68	–	–	–	[64]
						**FB2**	241	–	Fiber: ~ -72	–	–	–	
						**FB3**	51	–	Fiber: -100	–	–	–	
						**DON**	99	–	Fiber: -100	–	–	–	
						**ZEN**	68	–	Fiber: -100	–	–	–	
						**T-2**	24	–	Fiber: -100	–	–	–	

Experimental traditional West-African wet milling (incl. steeping/ fermentation of kernels in water for 72 h)	Maize (not further specified)	(not specified)	(not specified)	(not specified)	spiked	**FB1**	388	–	Fiber: ~ -81	–	–	–	
							794	–	Fiber: ~ -91	–	–	–	
					spiked	**DON**	396	–	Fiber: ~ -90	–	–	–	
							778	–	Fiber: ~ -85	–	–	–	
					spiked	**ZEN**	374	–	Fiber: ~ -73	–	–	–	
							762	–	Fiber: ~ -70	–	–	–	
					spiked	**T-2**	366	–	Fiber: ~ -83	–	–	–	
							749	–	Fiber: ~ -85	–	–	–	
Laboratory wet milling (incl. steeping of kernels in dilute solutions of lactic acid and SO_2, pH 4, at 49°C for 48 h)	Different batches of yellow maize	USA	(not specified)	1972	natural	**ZEN**	900	Gluten: ~ +656	Fiber: ~ +200	Germ: ~ +89	Solubles in steep water, wash water, filtrates: ~ +333	No loss/ degradation of ZEN	[65]
							4,100	Gluten: ~ +227	Fiber: ~ -12	Germ: ~ -12	Solubles: ~ +122		
							9,400	Gluten: ~ +117	Fiber: ~ -28	Germ: ~ -20	Solubles: ~ +13		
Commercial wet milling in a New Zealand facility (might include cleaning)	Maize (not further specified)	New Zealand	commercial (not further specified)	1993 or earlier	natural	**DON**	ca. 500	Gluten: ~ -80	Fiber: ~ -80	Germ: ~ -80	Animal feed (fiber + concentrated steep liquor): ~ -10	<u>Overall</u>: Very approximate data (mycotoxin levels were taken from diagrams)	[66]
	Maize (not further specified)	New Zealand	commercial (not further specified)	1993 or earlier	natural		ca. 450	Gluten: ~ -55	Fiber: ~ -60	Germ: ~ -55	Animal feed: ~ +450		
	Maize (not further specified)	USA	commercial (not further specified)	1993 or earlier	natural		ca. 200	(–)	(–)	(–)	Animal feed: ~ +220		
	Maize (not further specified)	New Zealand	commercial (not further specified)	1993 or earlier	natural	**ZEN**	ca. 250	Gluten: ~ +240	Fiber: ~ +100	Germ: ~ +180	Animal feed: ~ +50		

	Maize (not further specified)	New Zealand	commercial (not further specified)	1993 or earlier	natural		ca. 500	Gluten: ~ +1,300	Fiber: ~ +500	Germ: ~ +620	Animal feed: ~ +300		
Laboratory wet milling (incl. steeping of kernels in 1.5% lactic acid and 0.25% SO_2, pH 4, at 50–52°C for 48 h)	Different batches of yellow dent maize	(not specified)	(not specified)	(not specified)	post-harvest inoculation with an *Fusarium tricinctum* isolate	**T-2**	502	Gluten: ~ -12	Fiber: ~ -23	Germ: +94	Solubles: ~ +1,063	–	[67]
							225	Gluten: ~ -39	Fiber: ~ -39	Germ: +71	Solubles: ~ +856	–	
							8,700	Gluten: ~ +30	Fiber: ~ -12	Germ: +63	Solubles: ~ +814	–	
Experimental preliminary wet milling of steeped kernels (coarse grinding, density segregation in wash water and skim-off of germ) without further fractionation	Maize (not further specified)	(not specified)	(not specified)	(not specified)	post-harvest inoculation with an *Aspergillus ochraceus* isolate	**OTA**	~3,010	–	–	Germ: ~ -23	–	–	[68]

[a] Change in the mycotoxin concentration compared to the concentration in the unprocessed grain. Negative values: reduction; positive values: increase.

[b] It is assumed that the indicated initial values are on dry weight basis and that the indicated changes are corrected for changes in moisture content.

[c] In this study, the starch fraction was not collected since it could not be reliably related back to the a particular batch of input maize. But several starch samples from the processing facility, that had been analyzed at other times, showed nil or very low (close to detection limits) mycotoxin levels.

AF(s): aflatoxin(s) not further specified in the cited study; AFB1/2: aflatoxin B1/2; AFG1/2: aflatoxin G1/2; DON: deoxynivalenol; FB1/2/3: fumonisin B1/2/3; OTA: ochratoxin A; total AFs: aflatoxins B1+B2+G1+G2; total FBs: fumonisins B1+B2+B3; ZEN: zearalenone.

incl.: including

~: Approximate values that were calculated for this overview by using the data provided in the cited literature.

References

1. Visconti, A.; Haidukowski, E.M.; Pascale, M.; Silvestri, M. Reduction of deoxynivalenol during durum wheat processing and spaghetti cooking. *Toxicol. Lett.* **2004**, *153*, 181–189.
2. Ríos, G.; Zakhia-Rozis, N.; Chaurand, M.; Richard-Forget, F.; Samson, M.F.; Abecassis, J.; Lullien-Pellerin, V. Impact of durum wheat milling on deoxynivalenol distribution in the outcoming fractions. *Food Addit. Contam. Part A* **2009**, *26*, 487–495.
3. Herrera, M.; Juan, T.; Estopañan, G.; Ariño, A. Comparison of deoxynivalenol, ochratoxin A and aflatoxin B1 levels in conventional and organic durum semolina and the effect of milling. *J. Food Nutr. Res.* **2009**, *48*, 92–99.
4. Cheli, F.; Campagnoli, A.; Ventura, V.; Brera, C.; Berdini, C.; Palmaccio, E.; Dell'Orto, V. Effects of industrial processing on the distributions of deoxynivalenol, cadmium and lead in durum wheat milling fractions. *LWT-Food Sci. Technol.* **2010**, *43*, 1050–1057.
5. Brera, C.; Peduto, A.; Debegnach, F.; Pannunzi, E.; Prantera, E.; Gregori, E.; De Giacomo, M.; De Santis, B. Study of the influence of the milling process on the distribution of deoxynivalenol content from the caryopsis to cooked pasta. *Food Control* **2013**, *32*, 309–312.
6. L'vova, L.S.; Kizlenko, O.I.; Shul'gina, A.P.; Omel'chenko, M.D.; Bystryakova, Z.K. Distribution of deoxynivalenol in products of processing *Fusarium*-affected soft and hard wheats and barley. *Appl. Biochem. Microbiol.* **1998**, *34*, 444–449.
7. Samar, M.M.; Fontán, C.F.; Resnik, S.L.; Pacin, A.M.; Castillo, M. Distribution of deoxynivalenol in wheat, wheat flour, bran, and gluten, and variability associated with the test procedure. *J. AOAC Int.* **2003**, *86*, 551–556.
8. Edwards, S.G.; Dickin, E.T.; MacDonald, S.; Buttler, D.; Hazel, C.M.; Patel, S.; Scudamore, K.A. Distribution of *Fusarium* mycotoxins in UK wheat mill fractions. *Food Addit. Contam. Part A* **2011**, *28*, 1694–1704.
9. Giménez, I.; Herrera, M.; Escobar, J.; Ferruz, E.; Lorán, S.; Herrera, A.; Ariño, A. Distribution of deoxynivalenol and zearalenone in milled germ during wheat milling and analysis of toxin levels in wheat germ and wheat germ oil. *Food Control* **2013**, *34*, 268–273.
10. Young, J.C.; Fulcher, R.G.; Hayhoe, J.H.; Scott, P.M.; Dexter, J.E. Effect of milling and baking on deoxynivalenol (vomitoxin) content of eastern Canadian wheats. *J. Agric. Food Chem.* **1984**, *32*, 659–664.
11. Scott, P.M.; Kanhere, S.R.; Dexter, J.E.; Brennan, P.W.; Trenholm, H.L. Distribution of the trichothecene mycotoxin deoxynivalenol (vomitoxin) during the milling of naturally contaminated hard red spring wheat and its fate in baked products. *Food Addit. Contam.* **1984**, *1*, 313–323.
12. Lee, U.S.; Jang, H.S.; Tanaka, T.; Oh, Y.J.; Cho, C.M.; Ueno, Y. Effect of milling on decontamination of *Fusarium* mycotoxins nivalenol, deoxynivalenol and zearalenone in Korean wheat. *J. Agric. Food Chem.* **1987**, *35*, 126–129.
13. Tkachuk, R.; Dexter, J.E.; Tipples, K.H.; Nowicki, T.W. Removal by specific gravity table of tombstone kernels and associated trichothecenes from wheat infected with Fusarium head blight. *Cereal Chem.* **1991**, *68*, 428–431.
14. Nishio, Z.; Takata, K.; Ito, M.; Tanio, M.; Tabiki, T.; Yamauchi, H.; Ban, T. Deoxynivalenol distribution in flour and bran of spring wheat lines with different levels of Fusarium head blight resistance. *Plant Dis.* **2010**, *94*, 335–338.
15. Abbas, H.K.; Mirocha, C.J.; Pawlosky, R.J.; Pusch, D.J. Effect of cleaning, milling, and baking on deoxynivalenol in wheat. *Appl. Environ. Microbiol.* **1985**, *50*, 482–486.
16. Trigo-Stockli, D.M.; Deyoe, C.W.; Satumbaga, R.F.; Pedersen, J.R. Distribution of deoxynivalenol and zearalenone in milled fractions of wheat. *Cereal Chem.* **1996**, *73*, 388–391.
17. Zheng, Y.; Hossen, S.M.; Sago, Y.; Yoshida, M.; Nakagawa, H.; Nagashima, H.; Okadome, H.; Nakajima, T.; Kushiro, M. Effect of milling on the content of deoxynivalenol, nivalenol, and zearalenone in Japanese wheat. *Food Control* **2014**, *40*, 193–197.
18. Thammawong, M.; Okabe, M.; Kawasaki, T.; Nakagawa, H.; Nagashima, H.; Okadome, H.; Nakajima, T.; Kushiro, M. Distribution of deoxynivalenol and nivalenol in milling fractions from *Fusarium*-infected Japanese wheat cultivars. *J. Food Prot.* **2010**, *73*, 1817–1823.
19. Thammawong, M.; Okadome, H.; Shiina, T.; Nakagawa, H.; Nagashima, H.; Nakajima, T.; Kushiro, M. Distinct distribution of deoxynivalenol, nivalenol, and ergosterol in *Fusarium*-infected Japanese soft red winter wheat milling fractions. *Mycopathologia* **2011**, *172*, 323–330.

20. Kostelanska, M.; Dzuman, Z.; Malachova, A.; Capouchova, I.; Prokinova, E.; Skerikova, A.; Hajslova, J. Effects of milling and baking technologies on levels of deoxynivalenol and its masked form deoxynivalenol-3-glucoside. *J. Agric. Food Chem.* **2011**, *59*, 9303–9312.
21. Nowicki, T.W.; Gaba, D.G.; Dexter, J.E.; Matsuo, R.R.; Clear, R.M. Retention of the fusarium mycotoxin deoxynivalenol in wheat during processing and cooking of spaghetti and noodles. *J. Cereal Sci.* **1988**, *8*, 189–202.
22. Lancova, K.; Hajslova, J.; Kostelanska, M.; Kohoutkova, J.; Nedelnik, J.; Moravcova, H.; Vanova, M. Fate of trichothecene mycotoxins during the processing: Milling and baking. *Food Addit. Contam. Part A* **2008**, *25*, 650–659.
23. Banu, I.; Dragoi, L.; Aprodu, I. From wheat to sourdough bread: A laboratory scale study on the fate of deoxynivalenol content. *Qual. Assur. Saf. Crops* **2014**, *6*, 53–60.
24. Wang, L.; Shao, H.; Luo, X.; Wang, R.; Li, Y.; Li, Y.; Chen, Z. Effect of ozone treatment on deoxynivalenol and wheat quality. *PLoS ONE* **2016**, *11*, e0147613.
25. Young, J.C.; Subryan, L.M.; Potts, D.; McLaren, M.E.; Gobran, F.H. Reduction in levels of deoxynivalenol in contaminated wheat by chemical and physical treatment. *J. Agric. Food Chem.* **1986**, *34*, 461–465.
26. Tanaka, T.; Hasegawa, A.; Yamamoto, S.; Matsuki, Y.; Ueno, Y. Residues of *Fusarium* mycotoxins, nivalenol, deoxynivalenol and zearalenone, in wheat and processed food after milling and baking. *Food Hyg. Saf. Sci.* **1986**, *27*, 653–655.
27. Tibola, C.S.; Fernandes, J.M.C.; Guarienti, E.M.; Nicolau, M. Distribution of *Fusarium* mycotoxins in wheat milling process. *Food Control* **2015**, *53*, 91–95.
28. Tibola, C.S.; Fernandes, J.M.C.; Guarienti, E.M. Effect of cleaning, sorting and milling processes in wheat mycotoxin content. *Food Control* **2016**, *60*, 174–179.
29. Savi, G.D.; Piacentini, K.C.; Tibola, C.S.; Santos, K.; Sousa Maria, G.; Scussel, V.M. Deoxynivalenol in the wheat milling process and wheat-based products and daily intake estimates for the Southern Brazilian population. *Food Control* **2016**, *62*, 231–236.
30. Belluco, B.; de Camargo, A.C.; da Gloria, E.M.; Dias, C.T.d.S.; Button, D.C.; Calori-Domingues, M.A. Deoxynivalenol in wheat milling fractions: A critical evaluation regarding ongoing and new legislation limits. *J. Cereal Sci.* **2017**, *77*, 284–290.
31. Vaclavikova, M.; Malachova, A.; Veprikova, Z.; Dzuman, Z.; Zachariasova, M.; Hajslova, J. 'Emerging' mycotoxins in cereals processing chains: Changes of enniatins during beer and bread making. *Food Chem.* **2013**, *136*, 750–757.
32. Pascale, M.; Haidukowski, M.; Lattanzio, V.M.T.; Silvestri, M.; Ranieri, R.; Visconti, A. Distribution of T-2 and HT-2 toxins in milling fractions of durum wheat. *J. Food Prot.* **2011**, *74*, 1700–1707.
33. Scudamore, K.A.; Patel, S.; Edwards, S. HT-2 toxin and T-2 toxin in commercial cereal processing in the United Kingdom, 2004–2007. *World Mycotoxin J.* **2009**, *2*, 357–365.
34. Scudamore, K.A.; Banks, J.; MacDonald, S.J. Fate of ochratoxin A in the processing of whole wheat grains during milling and bread production. *Food Addit. Contam.* **2003**, *20*, 1153–1163.
35. Peng, C.; Wang, L.; An, F.; Zhang, L.; Wang, Y.; Li, S.; Wang, C.; Liu, H. Fate of ochratoxin A during wheat milling and some Chinese breakfast processing. *Food Control* **2015**, *57*, 142–146.
36. Njapau, H.; Muzungaile, E.M.; Changa, R.C. The effect of village processing techniques on the content of aflatoxins in corn and peanuts in Zambia. *J. Sci. Food Agric.* **1998**, *76*, 450–456.
37. Mutungi, C.; Lamuka, P.; Arimi, S.; Gathumbi, J.; Onyango, C. The fate of aflatoxins during processing of maize into *muthokoi*—A traditional Kenyan food. *Food Control* **2008**, *19*, 714–721.
38. Matumba, L.; Monjerezi, M.; Chirwa, E.; Lakudzala, D.; Mumba, P. Natural occurrence of AFB1 in maize and effect of traditional maize flour production on AFB1 reduction, in Malawi. *Afr. J. Food Sci.* **2009**, *3*, 413–425.
39. Castells, M.; Marín, S.; Sanchis, V.; Ramos, A.J. Distribution of fumonisins and aflatoxins in corn fractions during industrial cornflake processing. *Int. J. Food Microbiol.* **2008**, *123*, 81–87.
40. Pietri, A.; Zanetti, M.; Bertuzzi, T. Distribution of aflatoxins and fumonisins in dry-milled maize fractions. *Food Addit. Contam. Part A* **2009**, *26*, 372–380.
41. Romer, T. Detecting mycotoxins in corn and corn-milling products. *Feedstuffs* **1984**, *56*, 22–23.
42. Brera, C.; Catano, C.; de Santis, B.; Debegnach, F.; de Giacomo, M.; Pannunzi, E.; Miraglia, M. Effect of industrial processing on the distribution of aflatoxins and zearalenone in corn-milling fractions. *J. Agric. Food Chem.* **2006**, *54*, 5014–5019.
43. Brera, C.; Debegnach, F.; Grossi, S.; Miraglia, M. Effect of industrial processing on the distribution of fumonisin B1 in dry milling corn fractions. *J. Food Prot.* **2004**, *67*, 1261–1266.

44. Generotti, S.; Cirlini, M.; Dall'Asta, C.; Suman, M. Influence of the industrial process from caryopsis to cornmeal semolina on levels of fumonisins and their masked forms. *Food Control* **2015**, *48*, 170–174.
45. Vanara, F.; Reyneri, A.; Blandino, M. Fate of fumonisin B1 in the processing of whole maize kernels during dry-milling. *Food Control* **2009**, *20*, 235–238.
46. Vanara, F.; Scarpino, V.; Blandino, M. Fumonisin distribution in maize dry-milling products and by-products: Impact of two industrial degermination systems. *Toxins* **2018**, *10*, 357.
47. Scarpino, V. Fate of moniliformin during different large-scale maize dry-milling processes. *LWT-Food Sci. Technol.* **2020**, *123*, 109098.
48. Katta, S.K.; Cagampang, A.E.; Jackson, L.S.; Bullerman, L.B. Distribution of *Fusarium* molds and fumonisins in dry-milled corn fractions. *Cereal Chem. J.* **1997**, *74*, 858–863.
49. Broggi, L.E.; Resnik, S.L.; Pacin, A.M.; González, H.H.L.; Cano, G.; Taglieri, D. Distribution of fumonisins in dry-milled corn fractions in Argentina. *Food Addit. Contam.* **2002**, *19*, 465–469.
50. Bordini, J.G.; Ono, M.A.; Garcia, G.T.; Fazani, V.H.M.; Vizoni, É.; Rodrigues, K.C.B.; Hirooka, E.Y.; Ono, E.Y.S. Impact of industrial dry-milling on fumonisin redistribution in non-transgenic corn in Brazil. *Food Chem.* **2017**, *220*, 438–443.
51. Bordini, J.G.; Ono, M.A.; Garcia, G.T.; Vizoni, É.; Amador, I.R.; Hirozawa, M.T.; Ono, E.Y.S. Transgenic versus conventional corn: Fate of fumonisins during industrial dry milling. *Mycotoxin Res.* **2019**, *35*, 169–176.
52. Burger, H.M.; Shephard, G.S.; Louw, W.; Rheeder, J.P.; Gelderblom, W.C.A. The mycotoxin distribution in maize milling fractions under experimental conditions. *Int. J. Food Microbiol.* **2013**, *165*, 57–64.
53. Bryła, M.; Szymczyk, K.; Jędrzejczak, R.; Obiedziński, M.W. Free and hidden fumonisins in various fractions of maize dry milled under model conditions. *LWT-Food Sci. Technol.* **2015**, *64*, 171–176.
54. Aprodu, I.; Banu, I. Co-occurrence of fumonisins and T-2 toxins in milling maize fractions under industrial conditions. *CyTA—J. Food* **2015**, *13*, 102–106.
55. Schollenberger, M.; Müller, H.M.; Rüfle, M.; Suchy, S.; Drochner, W. Redistribution of 16 *Fusarium* toxins during commercial dry milling of maize. *Cereal Chem. J.* **2008**, *85*, 557–560.
56. Patey, A.L.; Gilbert, J. Fate of *Fusarium* mycotoxins in cereals during food processing and methods for their detoxification. In *Fusarium—Mycotoxins, Taxonomy, Pathogenicity*; Chelkowski, J., Ed.; Elsevier: Amsterdam, The Netherlands, 1989; Volume 2, pp. 399–420.
57. Aly, S.E. Distribution of aflatoxins in product and by-products during glucose production from contaminated corn. *Food/Nahr.* **2002**, *46*, 341–344.
58. Aly, S.E.; Hathout, A.S. Fate of aflatoxin B1 in contaminated corn gluten during acid hydrolysis. *J. Sci. Food Agric* **2011**, *91*, 421–427.
59. Yahl, K.R.; Watson, S.A.; Smith, R.J.; Barabolok, R. Laboratory wet-milling of corn containing high levels of aflatoxin and a survey of commercial wet-milling products. *Cereal Chem.* **1971**, *48*, 385–391.
60. Park, J.; Kim, D.-H.; Moon, J.-Y.; An, J.-A.; Kim, Y.-W.; Chung, S.-H.; Lee, C. Distribution analysis of twelve mycotoxins in corn and corn-derived products by LC-MS/MS to evaluate the carry-over ratio during wet-milling. *Toxins* **2018**, *10*, 319.
61. Okeke, C.A.; Ezekiel, C.N.; Sulyok, M.; Ogunremi, O.R.; Ezeamagu, C.O.; Šarkanj, B.; Warth, B.; Krska, R. Traditional processing impacts mycotoxin levels and nutritional value of *ogi*—A maize-based complementary food. *Food Control* **2018**, *86*, 224–233.
62. Kamimura, H. Removal of mycotoxins during food processing. *マイコトキシン (JSM Mycotoxins)* **1999**, *1999*, 88–94.
63. Bennett, G.A.; Richard, J.L.; Eckhoff, S.R. Distribution of fumonisins in food and feed products prepared from contaminated corn. In *Fumonisins in Food*; Jackson, L.S., DeVries, J.W., Bullerman, L.B., Eds.; Springer: Boston, MA, USA, 1996; pp. 317–322. https://doi.org/10.1007/978-1-4899-1379-1_27.
64. Chilaka, C.A.; De Boevre, M.; Atanda, O.O.; De Saeger, S. Fate of *Fusarium* mycotoxins during processing of Nigerian traditional infant foods (*ogi* and soybean powder). *Food Res. Int.* **2019**, *116*, 408–418.
65. Bennett, G.A.; Vandegraft, E.E.; Shotwell, O.L.; Watson, S.A.; Bocan, B.J. Zearalenone: Distribution in wet-milling fractions from contaminated corn. *Cereal Chem.* **1978**, *55*, 455–460.
66. Lauren, D.R.; Ringrose, M.A. Determination of the fate of three *Fusarium* mycotoxins through wet-milling of maize using an improved HPLC analytical technique. *Food Addit. Contam.* **1997**, *14*, 435–443.
67. Collins, G.J.; Rosen, J.D. Distribution of T-2 toxin in wet-milled corn products. *J. Food Sci.* **1981**, *46*, 877–879.
68. Wood, G.M. Effects of processing on mycotoxins in maize. *Chem. Ind.* **1982**, *18*, 972–974.
